# Synthesis and antiproliferative activity of pterostilbene and $3^\prime$-methoxy pterostilbene Mannich base derivatives against Hela cells

**DOI:** 10.1007/s11030-015-9615-1

**Published:** 2015-07-11

**Authors:** Chongyang Liu, Linpei Dong, Shengchun Wang, Qiuan Wang

**Affiliations:** College of Chemistry and Chemical Engineering, Hunan University, Changsha, 410082 People’s Republic of China

**Keywords:** Pterostilbene, $3^\prime$-Methoxy pterostilbene, Mannich base derivatives, Synthesis, Antiproliferative activity, Hela cells

## Abstract

**Abstract:**

Fourteen novel pterostilbene (**1**) and $3^\prime$-methoxy pterostilbene (**2**) Mannich base derivatives (**3**–**16**) were synthesized via the microwave-assisted Mannich reaction of **1** or **2** with various secondary amines and formaldehyde. The regioselectivity of the reaction occurred preferentially at $\hbox {C-3}^\prime (\hbox {and /or C-5}^\prime )$ position of the B-ring of stilbene. The biological testing results showed that all the target compounds exhibit antiproliferative activity against Hela cells from $\hbox {IC}_{50}=22.5$–$65.3\,\upmu \hbox {M}$. Compounds **1**–**3**, **7**, **11**–**13**, and **16** displayed higher (lower $\hbox {IC}_{50}$ values) activity than the positive control cisplatin $(\hbox {IC}_{50}= 41.3\,\upmu \hbox {M})$.

**Graphical Abstract:**

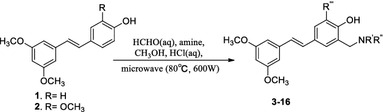

**Electronic supplementary material:**

The online version of this article (doi:10.1007/s11030-015-9615-1) contains supplementary material, which is available to authorized users.

## Introduction

Pterostilbene (*trans*-3,5-dimethoxy-$4^\prime$-hydroxystilbene, **1**), a naturally occurring dimethylether analog of resveratrol, was first isolated from *P. Santalinus* (red sandalwood) and it is also found in several types of berries and grapes [1]. Pterostilbene has been shown to have a cancer chemopreventive effect similar to that of resveratrol and it is cytotoxic to a variety of tumor cell types, such as human gastric carcinoma cells [2], human prostate cancer cells [3], and lung cancer cells [4]. $3^\prime$-Methoxy pterostilbene (**2**), a naturally occurring homolog of pterostilbene, was isolated from *Sphaerophysa salsula* [5] and showed higher cytotoxic activities on some cancer cells than resveratrol [6].

Biological activity studies have shown that the development of structural analogs of antitumor agents possessing basic nitrogen atom moiety is of great value [7, 8]. Anticancer studies using several classes of polyaromatic antitumour agents indicate that the introduction of a nitrogen-containing side chain increases significantly the biological activity and potency of the parent compounds. For example, it has been shown that the presence of the nitrogen moiety in flavopiridol, a synthetic nitrogen-containing flavonoid with antitumor activity against various tumor cell lines, is critical for its antitumor effect [9]. The Mannich reaction is a commonly used way to introduce nitrogenous moieties, such as aminoalkyl group to phenols. The modification of an aminoalkyl side chain in aromatic substrates permits to increase significantly the biological potency of bioactive molecules due to the greater number of molecular sites for electrophilic attack by cellular constituents, as well as due to the cascade effect of preferential chemosensitization when compared with parent compounds [10]. Furthermore, Mannich bases have been associated with increased water solubility [11].

Resveratrol has been the subject of numerous investigations, and its cancer chemopreventive activity has been fully demonstrated [12]. Prompted by the close structural similarity of pterostilbene and resveratrol, as well as the current high interest in the development of a pterostilbene-based therapeutic [13, 14], we decided to synthesize two series of fourteen pterostilbene and $3^\prime$-methoxy pterostilbene Mannich base derivatives to be evaluated for their antiproliferative activity against human cervical carcinoma Hela cells by the standard CCK-8 assay.

## Results and discussion

**Scheme 1:**
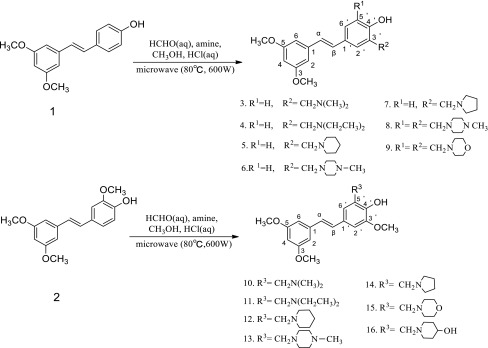
Synthesis of pterostilbene and $3^\prime$-methoxy pterostilbene Mannich base derivatives (**3**–**16**)

The synthetic route used for the construction of novel pterostilbene and $3^\prime$-methoxy pterostilbene Mannich base derivatives (**3**–**16**) is shown in Scheme 1. Pterostilbene (**1**) and $3^\prime$-methoxy pterostilbene (**2**) were synthesized by *O*-methylation, reduction, chlorination, Arbuzov rearrangement, and Wittig reaction using commercially available 3,5-dihydroxybenzoic acid, 4-hydroxybenzaldehyde, or vanillin as starting material as described previously by our group [15].

Our strategy for the synthesis of the *C*-aminomethylated derivatives relied upon the electrophilic substitution at $\hbox {C-3}^\prime$ and/or $\hbox {C-5}^\prime$ of the stilbene B-ring. This was achieved by the Mannich reaction of **1** and **2** with formaldehyde and secondary amines in methanol, introducing a dialkylaminomethyl group in the *ortho-* position of the phenolic group. The general conditions of the Mannich reaction for the phenol compounds are based on the substrate, the amine, and formaldehyde ratio in alcohol with prolonged heating. It was reported that microwave irradiation can be used for the Mannich reaction because of the considerable advantages over conventional heating, such as substantial rate enhancement cleaner reaction and improvement in yield [16, 17]. In our case, pterostilbene (**1**) or $3^\prime$-methoxypterostilbene (**2**), formaldehyde, and secondary amines in a 1:1.2:1.2 ratio, respectively, were stirred under microwave irradiation (600 W) for 0.5–2 h in methanol to afford the *C*-aminomethylated derivatives **3**–**16**. The Mannich reaction of pterostilbene (**1**) with methylpiperazine would lead to a different product at different reaction times where the product was a mono-substituted product (compound **6**, 0.5 h of reaction time) or a double-substituted product (compound **8**, 2 h of reaction time).

The structures of all synthesized Mannich base were confirmed by $^{1}\hbox {HNMR}, ^{13}\hbox {CNMR}$, and MS analysis. The $^{1}\hbox {HNMR}$ spectra of compounds **3**–**9** clearly indicate the absence of the of $\hbox {H-3}^\prime$ proton signal at $\delta \,6.28$ (and $\hbox {H-5}^\prime$ proton for compounds **8**, **9**) in the B-ring of pterostilbene (**1**). Similarly, the $\hbox {H-5}^\prime$ proton at $\delta \,6.91$ of $3^\prime$-methoxy pterostilbene (**2**) in the B-ring disappeared for compounds **10**–**16**. The signal at $\delta$ 3.60–4.10 indicated the presence of the aminomethyl group at $\hbox {C-3}^\prime \,(\hbox {or C-5}^\prime )$ position of compounds **3**–**16**.

**Fig. 1:**
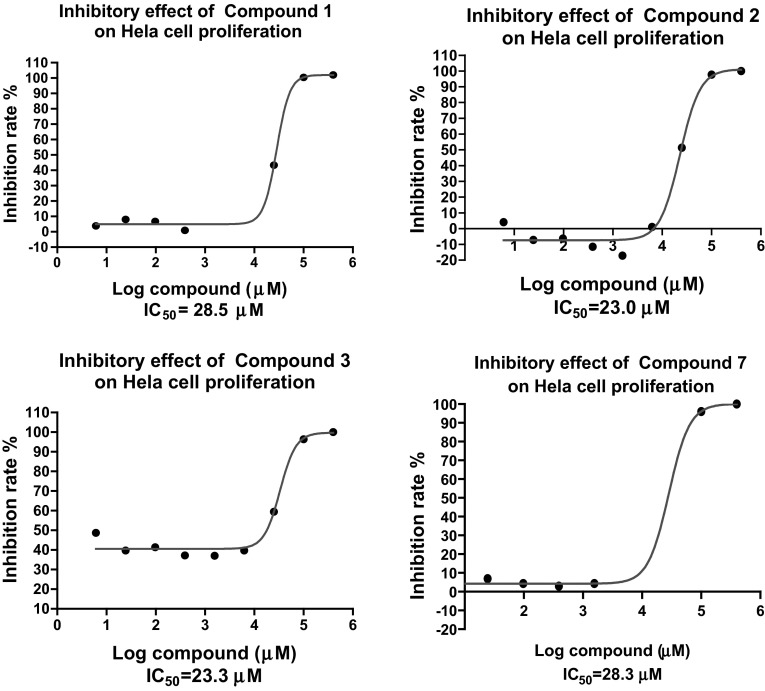
Dose-response curve of compounds **1**, **2**, **3 ** and **7** on Hela cell proliferation assay

The antiproliferative activity of the pterostilbene (**1**), $3^\prime$-methoxypterostilbene (**2**), and the Mannich base derivatives **3**–**16** was assessed using the CCK-8 [2H-tetrazolium, 5-(2,4-disulfophenyl)-3-(2-methoxy-4-nitrophenyl)-2-(4- nitrophenyl) inner salt, sodium salt (1:1)] assay using the human cervical carcinoma Hela cell line. The results are shown in Table 1. The dose-response curves for the CCK-8 assay of compounds **1**, **2**, **3 ** and **7 ** on Hela cells proliferation are shown in Fig. 1. Cisplatin (DDP) was used as positive control. The results show that all the test compounds exhibit moderate to potent antiproliferative activity against Hela cells.

**Table 1:** Half-inhibitory concentration $[ \hbox {IC}_{50}(\upmu \hbox {M})]$ of compounds **1**–**16** on the Hela cells

Compounds	$\hbox {IC}_{50 }(\upmu \hbox {M})$	Compounds	$\hbox {IC}_{50 }(\upmu \hbox {M})$
**1**	28.5	**10**	41.6
**2**	23.0	**11**	35.4
**3**	23.3	**12**	38.4
**4**	41.8	**13**	387
**5**	42.5	**14**	43.0
**6**	50.4	**15**	65.3
**7**	28.3	**16**	36.7
**8**	57.1	cisplatin$^\mathrm{a}$ (DDP)	41.3
**9**	50.5		

$^\mathrm{a}$ cisplatin (DDP) was employed as positive control

In the series of pterostilbene Mannich bases, our data showed that compound **3**, characterized by a dimethylaminomethyl group in the $\hbox {C-3}^\prime$ position, was found to have an increase in antiproliferative activity $(\hbox {IC}_{50} = 23.3\,\upmu \hbox {M})$ when compared with parent compound pterostilbene $(\mathbf{1}, \hbox {IC}_{50} = 28.5\,\upmu \hbox {M})$. Compound **7 **$(\hbox {IC}_{50} = 28.3\,\upmu \hbox {M})$, which contains a pyrrolidin-1-ylmethyl group substituent on the $\hbox {C-3}^\prime$ position, presented with potency similar to that of parent compound pterostilbene (**1**). Compounds **1**, **3**, and **7 **$(\hbox {IC}_{50}$ range 23.3–28.5 $\upmu \hbox {M})$ showed more potent antiproliferative activity against Hela cells than the positive control cisplatin (DDP) $(\hbox {IC}_{50} = 41.3\, \upmu \hbox {M})$. In the series of $3^\prime$-methoxypterostilbene Mannich bases, although the introduction of a dialkylaminomethyl group did not increase the activity compared to its parent compound **2**, compounds **11**, **12**, **13** and **16 ** exhibited some antiproliferative activity on Hela cells $(\hbox {IC}_{50}$ range of 35.4–38.7 $\upmu \hbox {M})$ being slightly more potent than cisplatin (DDP) $(\hbox {IC}_{50}= 41.3\,\upmu \hbox {M})$.

The preliminary structure–activity relationship analysis revealed that pterotilbene (**1**) has a moderate antiproliferative activity on Hela cells. The introduction of smaller methoxy or dimethylaminomethyl side chains into the 3$^\prime$-position of **1** was to increase biological activity, and the introduction of larger dialkylaminomethyl side chain into 3$^\prime$-position of **1** was to decrease the activity. Based on these observations, the methoxy and dimethylaminomethyl groups were selected as suitable substituents at the 3$^\prime$-position of **1**. The introduction of all dialkylaminomethyl side chain into 5$^\prime$-position of 3$^\prime$-methoxy pterostilbene (**2**) was to decrease the activity. As the antiproliferation assay was carried out in vitro, the inhibitory effect on Hela cell variance among these compounds could be attributed to the hydrophobicity and permeability changes resulting from different substituents and position of side chains in pterostilbene (**1**) .

## Conclusion

In Summary, 14 new pterostilbene (**1**) and $3^\prime$-methoxy pterostilbene (**2**) Mannich base derivatives **3**–**16** were synthesized through a microwave-assisted Mannich reaction strategy. All the compounds were tested for their antiproliferative activity against Hela cells using the standard CCK-8 assay; the results indicating that compounds **1**, **2**, **3**, **7**, **11**, **12**, **13 ** and **16** were slightly more potent than anticancer drug cisplatin (DDP). The pterostilbene skeleton can serve as a promising structural template for the development of novel antiproliferative agents deserving further investigation.

## Experimental

### General information

Melting points were measured on an XRC-I apparatus and were uncorrected. Microwave reactions were performed using an XH-MC-1 microwave reactor (50–900 W) (Beijing Xianghu Science and Technology Development Co., China). $^{1}\hbox {H}$ NMR and $^{13}\hbox {C}$ NMR spectra were recorded on a Bruker AM-400 instrument, using tetramethylsilane as internal standard (*s* single; *d* doublet; *t* triplet; *q* quarlet; *m* multiplet), chemical shifts $(\delta )$ in ppm, and coupling constants (*J*) in Hz. Mass spectra were recorded using a VG Autospec-3000 and a ZA-BHS spectrometer (ESI or EI ionization method). Pterostilbene (**1**) and $3^\prime$-methoxy pterostilbene (**2**) were synthesized and purified according to our published procedure [15]. Formaldehyde, secondary amines, methanol, ethylacetate, petroleum ether, triethylamine, CCK-8 [2H-tetrazolium, 5-(2,4-disulfophenyl)-3-(2-methoxy-4-nitrophenyl)-2-(4-nitrophenyl) inner salt, sodium salt (1:1)] were obtained from Energy Chemicals. Column chromatography was carried out on silica gel (200–300 mesh). Commercially available AR grade or chemically pure reagents were used. Anhydrous solvents were dried and redistilled prior to their use.

### General procedure for the synthesis of Mannich base derivatives 3–16

Compound **1** or **2** (200 mg, 0.78 mmol) was dissolved in methanol (10 mL), then formaldehyde (0.94 mmol) and a secondary amine (0.94 mmol) were introduced to this solution. The reaction mixture was stirred under microwave irradiation (600 W) at $80\,^ {\circ }\hbox {C}$. The end of the reaction was determined by TLC. When the reaction was complete, the solvent was removed in *vacuo.* The residue was extracted with ethyl acetate $(3\times 20\,\hbox {mL})$ and water (15 mL). The organic phases were combined, washed with $\hbox {H}_{2}\hbox {O}$, and dried over anhydrous sodium sulfate. The solvent was evaporated and resulting crude material was purified by silica gel column chromatography (ethyl acetate:petroleum ether:triethylamine, *v* / *v*, 1:9:0.01) to obtain desired products **3**–**16**.

#### $(E){\text {-}}3^\prime$*-[(Dimethylamino)methyl]pterostilbene* (**3**)

**3** was obtained as yellow oil (169 mg, yield: 69 %). $^{1}\hbox {H}\,\hbox {NMR }\,(\hbox {400 MHz}, \hbox {CDCl}_{3}){:} \delta \,9.83 (\hbox {s}, \hbox {1H}, \hbox {OH})$7.23 $(\hbox {d}, \hbox {J} = 8.2\,\hbox {Hz}, \hbox {1H}, 6^\prime \hbox {-H}),\, 7.03 (\hbox {s}, \hbox {1H}, 2^\prime \hbox {-H}),\, 6.90 \,(\hbox {d}, \hbox {J} = 16.2\,\hbox {Hz}, \hbox {1H}, \beta \hbox {-CH=}), 6.76 (\hbox {d}, \hbox {J} \!=\! 16.2\,\hbox {Hz}, \hbox {1H}, \alpha \hbox {-CH=}), 6.73 (\hbox {d}, \hbox {J} = 8.6\,\hbox {Hz}, \hbox {1H}, 5^\prime \hbox {-H})., 6.54 (\hbox {s}, \hbox {2H}, \hbox {2-H and 6-H}), 6.27 \,(\hbox {s, 1H,4-}\hbox {H}), 3.71 (\hbox {s, 6H}, \hbox {3-OCH}_{3}\hbox { and }\hbox {5-OCH}_{3}), 3.55 (\hbox {s, 2H}, 3^\prime {-}\hbox {CH}_{2}\hbox {N}), 2.23 (\hbox {s, 6H}, \hbox {N}(\hbox {CH}_{3})_{2}); ^{13}\hbox {C NMR }(100\hbox { MHz}, \hbox {CDCl}_{3}): \delta 161.0,\! 158.3,\! 139.9,\! 129.0,\! 128.3,\! 127.3,\! 126.7,\! 125.8,\! 122.1, 116.4, 104.3, 99.4, 62.8, 55.3, 44.4; \hbox { ESIMS}\, m/z{:} 314\, [\hbox {M}+\hbox {H}]^{+}$.

#### $(E){\text {-}}3^\prime$*-[(Diethylamino)methyl]pterostilbene* (**4**)

**4 ** was obtained as yellow oil (168 mg, yield 63 %). $^{1}\hbox {H NMR} \,(\hbox {400\,MHz, CDCl}_{3}){:}\;\delta \,7.22 \,(\hbox {d}, J = 8.3\hbox { Hz, 1H}, 6'\hbox {-H}),\, 7.03 \,(\hbox {s, 1H}, 2'\hbox {-H}),\, 6.90 \,(\hbox {d}, J = 16.2\hbox { Hz, 1H}, \beta \hbox {-CH}=),\, 6.76 \,(\hbox {d}, J = 16.2\hbox { Hz, 1H}, \alpha \hbox {-CH=}),\, 6.70 \,(\hbox {d}, J = 8.3\hbox { Hz, 1H}, 5'\hbox {-H}), 6.54 (\hbox {d, 2H, 2-H and 6-H}), 6.27 (\hbox {s, 1H,}\hbox {4-H}),\, 3.71 \,(\hbox {s, 6H, 3-OCH}_{3 }\hbox { and 5-OCH}_{3}),\, 3.68\, \,(\hbox {s, 2H}, 3^\prime \hbox {-CH}_{2}\hbox {N}), 2.53 (\hbox {q}, J = 6.8\hbox { Hz, 4H, 2CH}_{2}),\, 1.01 \,(\hbox {t}, J = 7.0\hbox { Hz, 6H,} \hbox {2CH}_{3}). ^{13}\hbox {C NMR }(\hbox {100 MHz, CDCl}_{3}): \delta 161.0, 158.6 140.0, 129.1, 128.2, 127.0, 126.7, 125.6, 122.3, 116.4, 104.3,\! 99.4, 56.9, 55.3, 46.3, 11.2;\hbox { ESIMS } m/z\, 342\hbox { [M+H]}^{+}$.

#### $(E){\text {-}}3^\prime$*-(Piperidin-1-ylmethyl)pterostilbene* (**5**)

**5 ** was obtained as white crystals (182 mg, yield 66 %), $\hbox {m.p.}\;80{-}81\,^{\circ }\hbox {C}. ^{1}\hbox {H NMR }(400\hbox { MHz}, \hbox {CDCl}_{3}){:}\; \delta \,7.35 \,(\hbox {d}, J = 7.6\,\hbox { Hz}, 6^\prime \hbox {-H}),\, 7.15 \,(\hbox {s, 1H}, 2^\prime \hbox {-H}),\, 7.00 (\hbox {d}, J = 16.2\,\hbox { Hz,} \hbox {1H}, \beta \hbox {-CH=}),\, 6.86 \; (\hbox {d}, J = 16.2\,\hbox { Hz, 1H}, \alpha \hbox {-CH=}),\, 6.82 (\hbox {s,} \hbox {1H}, 5^\prime \hbox {-H}),\, 6.64 \,(\hbox {s,}\hbox {2H, 2-H and 6-H}),\, 6.37 \,(\hbox {d}, J = 2.1\,\hbox {Hz,} \hbox {1H, 4-H}),\, 3.83 \,(\hbox {s, 6H, 3-OCH}_{3} \hbox { and 5-OCH}_{3}),\, 3.73 \,(\hbox {s}, \hbox {2H}, 3^\prime \hbox {-CH}_{2}\hbox {N}),\, 2.57 \,(\hbox {t}, J \!=\! 2.3\,\hbox { Hz, 4H, NCH}_{2}),\, 1.69{-}1.65 (\hbox {m}, J \!=\! 5.0\,\hbox {Hz, 4H, 2CH}_{2}), 1.55{-}1.52 \,(\hbox {m, J = 1.6\,Hz,} \hbox {2H},\hbox {CH}_{2}); ^{13}\hbox {C NMR }(\hbox {100 MHz, CDCl}_{3}): \delta 159.9, 157.2,138.8, 128.0, 127.1, 126.0,\, 125.7,\, 124.6,\, 120.7, 115.3, 103.1, 98.3, 61.1, 54.3, 52.8, 24.8, 22.9; \hbox { ESIMS } m/z: \hbox { 354 [M+H]}^{+}$.

#### $(E){\text {-}}3^\prime$*-[(4-Methylpiperazin-1-yl)methyl]pterostilbene* (**6**)

**6 ** was obtained as yellow oil (184 mg, yield 64 %). $^{ 1}\hbox {H NMR} \,(\hbox {400 MHz, CDCl}_{3}){:}\;\delta 7.26 \,(\hbox {d}, J \!=\! 8.3\,\hbox { Hz, 1H}, 6^\prime \hbox {-H}),\, 7.07 \,(\hbox {s, 1H}, 2^\prime \hbox {-H}), 6.91 (\hbox {d}, J \!=\! 16.2\,\hbox { Hz, 1H,}\,\beta \hbox {-CH=}),6.82 \,(\hbox {d}, J \!=\! 16.2\,\hbox { Hz, 1H}, \alpha \hbox {-CH=}), 6.74 (\hbox {d}, J \!=\! 7.9\,\hbox { Hz, 1H}, 5^\prime \hbox {-H}),\, 6.56 \,(\hbox {s, 2H, 2-H and 6-H}),\, 6.29 \,(\hbox {s, 1H,} \hbox {4-H}),\, 3.74 \,(\hbox {s,} \hbox {6H, 3-}\hbox {OCH}_{3}\hbox { and 5-OCH}_{3}), 3.66 \!(\hbox {s, 2H}, 3^\prime \hbox {-CH}_{2}\hbox {N}), 2.80\!-\! 2.27 (\hbox {m},~\hbox {8H, NCH}_{2}),\, 2.25 \,(\hbox {s, 3H, NCH}_{3});\,^{ 13}\hbox {C NMR } (\hbox {100 MHz}, \hbox {CDCl}_{3}): \delta 159.9, 156.7, 138.7, 127.8, 127.5,\, 126.3,\, 125.9, 124.9, 120.2, 115.4, 103.2, 98.4, 60.3, 54.3, 53.8, 51.3,\! 44.8; \hbox {ESIMS }m/z{:}\hbox { 369 [M+H]}^{+}$.

#### $(E){\text {-}}3^\prime$*-(Pyrrolidin-1-ylmethyl) pterostilbene* (**7**)

**7 ** was obtained as yellow oil (164 mg, yield 62 %). $^{1}\hbox {H NMR} \,(\hbox {400 MHz, CDCl}_{3}){:} \delta \,9.53 \,(\hbox {s, 1H, OH}),\, 7.35 \,(\hbox {d}, J =\! 8.3, 1.8\,\hbox { Hz, 1H},6^\prime \hbox {-H}),\, 7.18 \,(\hbox {d}, J \!=\! 1.3\,\hbox { Hz, 1H}, 2^\prime \hbox {-H}),\, 7.02 \,(\hbox {d}, J = 16.2\hbox { Hz, 1H}, \beta \hbox {-CH=}),\, 6.89 \,(\hbox {d}, J = 16.2\hbox { Hz,} \hbox {1H}, \alpha \hbox {-CH=}),\, 6.85 \,(\hbox {d}, J =\hbox { 8.4Hz, 1H}, 5^\prime \hbox {-H}),\, 6.66 \,(\hbox {d}, J = 2.1\,\hbox { Hz, 2H, 2-H and 6-H}),\, 6.39 \,(\hbox {d}, J = 2.0\,\hbox { Hz, 1H, 4-H}),\, 3.87 \,(\hbox {d}, J = 6.3\,\hbox { Hz, 2H}, 3^\prime \hbox {-CH}_{2}\hbox {N}),\, 3.84 (\hbox {s, 6H, 3-OCH}_{3}\hbox { and 5-OCH}_{3}),\, 2.69 \,(\hbox {s, 4H, N}(\hbox {CH}_{2})_{2}),\, 1.88 \,(\hbox {s, 4H, 2CH}_{2}); ^{13}\hbox {C NMR }(\hbox {100 MHz, CDCl}_{3}){:} \delta \,160.8, 158.1, 139.8, 128.9, 128.1, 127.1, 126.3, 125.6, 122.3, 116.3, 104.1, 99.3, 58.5, 55.3, 53.4, 23.6; \hbox {ESIMS } m/z{:}\hbox { 340 [M+H]}^{+}$.

#### $(E){\text {-}}3^\prime ,5^\prime$*-Bis[(4-methylpiperazin-1-yl)methyl] pterostilbene* (**8**)

**8 ** was obtained as yellow oil (131 mg, yield 35 %). $^{1}\hbox {H NMR} \,(\hbox {400 MHz, CDCl}_{3}){:} \delta \,7.18 \,(\hbox {s, 2H}, 2^\prime \hbox {-H and }6^\prime \hbox {-H}),\, 6.96 \,(\hbox {d}, J = 16.2\,\hbox { Hz, 1H}, \beta \hbox {-CH=}),\, 6.83 \,(\hbox {d}, J = 16.2\,\hbox { Hz,} \hbox {1H}, \alpha \hbox {-CH=}),\, 6.60 \,(\hbox {d}, J = 1.9\,\hbox { Hz, 2H, 2-H and 6-H}),\, 6.32 \,(\hbox {d}, J = 1.7 \hbox { Hz, 1H, 4-H}), 3.78 (\hbox {s, 6H, 3-OCH}_{3}\hbox { and}\hbox {5-}\hbox {OCH}_{3}),\, 3.62 (\hbox {d}, J \!=\! 4.3\hbox { Hz, 4H}, 3^\prime \hbox {-CH}_{2}\hbox {N and }5^\prime \hbox {-CH}_{2}\hbox {N}),\, 2.89{-}2.27 \,(\hbox {m, 16H, NCH}_{2}),\, 2.25 \,(\hbox {s, 6H, NCH}_{3});^{13}\hbox {C NMR }(\hbox {100} \hbox {MHz,}\,\hbox {CDCl}_{3}): \delta \, 160.8, 156.6, 139.8, 129.0, 127.7, 127.2, 125.7,\! 122.7,\! 104.1,\! 99.4,\! 58.8,\! 55.3,\! 54.9, 52.7, 45.9; \hbox {ESIMS} m/z{:}\, 481\hbox { [M+H]}^{+}$.

#### $(E){\text {-}}3^\prime ,5^\prime$*-Bis(morpholinomethyl)pterostilbene* (**9**)

**9 ** was obtained as yellow oil (117 mg, yield 33 %). $^{1}\hbox {H NMR} \,(\hbox {400 MHz, CDCl}_{3}){:} \delta \,7.17 \,(\hbox {s, 2H}, 2^\prime \hbox {-H and }6^\prime \hbox {-H}),\, 6.92 \,(\hbox {d}, J = 16.2\,\hbox { Hz,} \hbox {1H}, \beta \hbox {-CH=}),\, 6.80 \,(\hbox {d}, J = 16.2\,\hbox { Hz}, \hbox {1H}, \alpha \hbox {-CH=}),\, 6.56 \,(\hbox {s, 2H, 2-H and 6-H}),\, 6.29 \,(\hbox {s, 1H, 4-H}), 3.74 \,(\hbox {s, 6H, 3-OCH}_{3}\hbox { and 5-OCH}_{3}), 3.67 (\hbox {s, 8H}, \hbox {OCH}_{2}), 3.58 (\hbox {s, 4H}, 3^\prime \hbox {-CH}_{2}\hbox {N \,and }\,5^\prime \hbox {-CH}_{2}\hbox {N}),\, 2.49 \,(\hbox {s, 8H},\, \hbox {NCH}_{2});\, ^{13}\hbox {C} \hbox { NMR }(\hbox {100 MHz, CDCl}_{3}):\delta 161.0, 156.2, 139.7, 128.8, 128.1, 127.7, 126.1,\, 122.3,\, 104.3,\, 99.5,\, 66.8,\, 59.3,\, 55.4, 53.2; \hbox {ESIMS }m/z{:}\, 455 \hbox { [M+H]}^{+}$.

#### $(E){\text {-}}3^\prime$*-Methoxyl-5*$^\prime$*-((dimethylamino)methyl)pterostilbene* (**10**)

**10** was obtained as yellow oil (173 mg, yield 72 %). $^{1}\hbox {H NMR }(\hbox {400 MHz, CDCl}_{3}){:}\;\delta \,8.63 \,(\hbox {s, 1H, OH}),\, 6.99 \,(\hbox {s,} \hbox {1H}, 6^\prime \hbox {-H}),\, 6.97 \,(\hbox {d}, J \!=\! 16.2\hbox { Hz, 1H}, \beta \hbox {-CH=}),\, 6.86 \,(\hbox {d}, J = 16.2 \hbox {Hz, 1H}, \alpha \hbox {-CH=}),\, 6.75 \,(\hbox {s, 1H}, 2^\prime \hbox {-H}),\, 6.64 \,(\hbox {d}, J = \hbox {2.1} \hbox {Hz, 2H, 2-H and 6-H}),\, 6.36 \,(\hbox {d}, J \!=\! \hbox {2.1 Hz, 1H, 4-H}),\, 3.93 \,(\hbox {s, 3H}, 3^\prime \hbox {-OCH}_{3}),\, 3.81 \,(\hbox {s, 6H, 3-OCH}_{3}\hbox { and 5-OCH}_{3}), 3.66\,(\hbox {s, 2H}, 5^\prime \hbox {-CH}_{2}\hbox {N}), 2.34 (\hbox {s, 6H, NCH}_{3});^{ 13}\hbox {C NMR }(\hbox {100} \hbox {MHz,} \hbox {CDCl}_{3}): \delta \,160.8, 148.0, 147.6, 139.6, 129.1, 127.9, 125.8, 121.9,\, 119.4,\, 108.7,\, 104.1,\, 99.4,\, 62.5,\, 55.8,\, 55.2,\, 44.4;\hbox {EIMS }m/z{:}\, 343\hbox { [M]}^{+}$.

#### $(E){\text {-}}3^\prime$*-Methoxyl-5*$^\prime$*- ((diethylamino)methyl) pterostilbene* (**11**)

**11 **was obtained as yellow oil (189 mg, yield 73 %). $^{1}\hbox {H NMR }(\hbox {400 MHz, CDCl}_{3}): \delta \,6.90 \,(\hbox {s, 1H}, 6^\prime \hbox {-C}),\, 6.89 (\hbox {d}, J = 16.2\,\hbox { Hz}, \beta \hbox {-CH=}),\, 6.78 \,(\hbox {d, 1H}, J = \hbox {16.2 Hz},\alpha \hbox {-CH=}), 6.67 \,(\hbox {s, 1H}, 2^\prime \hbox {-H}),\, 6.56 (\hbox {d}, J = \hbox {2.1 Hz, 2H, 2-H}~\hbox {and 6-H}), 6.29 (\hbox {t}, J = \hbox {2.1 Hz, 1H,4-H}),\, 3.85 \,(\hbox {s, 3H}, 3^\prime ~\hbox {-OCH}_{3}),\, 3.74 (\hbox {s, 6H, 3-OCH}_{3}\hbox { and 5-OCH}_{3}),\, 3.71 \,(\hbox {s, 2H}, 5^\prime \hbox {-CH}_{2}\hbox {N}), 2.56 (\hbox {q}, J \!=\! \hbox {6.1 Hz, 4H, NCH}_{2}), 1.04 \,(\hbox {t}, J \!=\! \hbox {6.2 Hz, 6H, 2CH}_{3}); ^{ 13}\hbox {C NMR }(\hbox {100 MHz, CDCl}_{3})\!:\! \delta 160.8, 148.4, 147.9, 139.6, 129.4, 127.8, 125.8, 121.5, 119.6, 108.4, 104.1, 99.4, 56.0, 55.7, 55.2, 46.2, 11.0;\hbox { EIMS } m/z:371\hbox { [M]}^{+}$.

#### $(E){\text {-}}3^\prime$*-Methoxyl-5*$^\prime$*-(piperidin-1-yl methyl) pterostilbene* (**12**)

**12 **was obtained as yellow oil (185 mg, yield 69 %). $^{1}\hbox {H NMR }(\hbox {400 MHz, CDCl}_{3}){:} \delta \,6.90 \,(\hbox {s, 1H}, 6^\prime \hbox {-H}),\, 6.89 \,(\hbox {d}, J = \hbox {16.2 Hz}, \beta \hbox {-CH=}),\, 6.77 \,(\hbox {d}, J \!=\! \hbox {16.2 Hz, 1H}, \alpha \hbox {-CH=}),\, 6.65 \,(\hbox {d}, J = \hbox {1.5 Hz, 1H}, 2^\prime \hbox {-H}),\, 6.56 \,(\hbox {d}, J = \hbox {2.2 Hz, 2H,} \hbox {2-H and 6-H}),\, 6.28 \,(\hbox {d}, J = \hbox {2.2 Hz, 1H, 4-H}),\, 3.84 \,(\hbox {s, 3H}, 3^\prime \hbox {-OCH}_{3}),\, 3.72 \,(\hbox {s, 6H, 3-OCH}_{3}\hbox { and 5-OCH}_{3}),\, 3.59 \,(\hbox {s, 2H}, 5^\prime \hbox {-CH}_{2}\hbox {N}),\, 2.60-2.26 \,(\hbox {m, 4H,} \hbox {NCH}_{2}),\, 1.59{-}1.52 \,(\hbox {m, 4H,} \hbox {CH}_{2}),\, 1.40 \,(\hbox {s, 2H, CH}_{2});\, ^{13}\hbox {C NMR }(\hbox {100 MHz}, \hbox {CDCl}_{3}): \delta \,160.8,\, 147.9,\, 147.6,\, 139.6,\, 129.6,\, 127.7,\, 125.6,\, 121.4, 119.4, 108.4, 104.0, 99.3, 61.8, 55.7, 55.4, 537, 25.7, 23.8; \hbox { EIMS }m/z{:}\, 383\hbox { [M]}^{+}$.

#### $(E){\text {-}}3^\prime$*-Methoxyl-5*$^\prime$*-[(4-methylpiperazin-1-yl)methyl] pterostilbene* (**13**)

**13 ** was obtained as yellow oil (173 mg, yield 62 %). $^{1}\hbox {H NMR }(\hbox {400 MHz, CDCl}_{3}){:}\delta \,6.99(\hbox {s, 1H}, 6^\prime \hbox {-H}),\, 6.98 \,(\hbox {d}, J = \hbox {16.2 Hz, 1H},\beta \hbox {-CH=}),\, 6.86 \,(\hbox {d}, J = \hbox {16.2 Hz, 1H}, \alpha \hbox {-CH=}),\, 6.78 \,(\hbox {d}, J = \hbox {1.4 Hz, 1H}, 2^\prime \hbox {-H}),\, 6.64 \,(\hbox {d}, J = \hbox {2.2 Hz, 2H, 2-H and 6-H}),\, 6.37 \,(\hbox {t}, J = \hbox {2.2 Hz, 1H, 4-H}),\, 3.94 \,(\hbox {s, 3H}, 3^\prime \hbox {-OCH}_{3}),\, 3.83 \,(\hbox {s, 6H, 3-OCH}_{3}\hbox { and 5-OCH}_{3}),\, 3.75 \,(\hbox {s, 2H}, 5^\prime \hbox {-CH}_{2}\hbox {N}),\, 2.88{-}2.35 \,(\hbox {m, 8H, NCH}_{2}),\, 2.32 \,(\hbox {s}, \hbox {3H, NCH}_{3});^{ 13}\hbox {C NMR} \,(\hbox {100 MHz, CDCl}_{3}): \delta \,160.9, 148.1, 147.2,\, 139.7,\, 129.1,\, 128.2,\, 126.1,\, 121.1,\, 119.6,\, 108.7, 104.4, 99.5, 61.0, 55.9, 55.3, 54.8, 52.3, 45.7;\hbox { EIMS } m/z: 398\hbox { [M]}^{+}$.

#### $(E){\text {-}}3^\prime$*-Methoxyl-5*$^\prime$*-(pyrrolidin-1-ylmethyl) pterostilbene* (**14**)

**14 **was obtained as yellow oil (173 mg, yield 67 %). $^{1}\hbox {H NMR } \,(\hbox {400 MHz, CDCl}_{3}):\delta \,6.90 \,(\hbox {s, 1H}, 6^\prime \hbox {-H}),\, 6.89 (\hbox {d}, J = \hbox {16.2 Hz}, \beta \hbox {-CH=}),\, 6.78 \,(\hbox {d}, J = \hbox {16.2 Hz, 1H},\alpha \hbox {-CH=}),\, 6.68 \,(\hbox {s, 1H}, 2^\prime \hbox {-H}),\, 6.56\, (\hbox {d}, J = \hbox {2.1 Hz, 2H, 2-H}\, \hbox {and 6-H}),\, 6.29 \,(\hbox {s, 1H, 4-H}),\, 3.85 \,(\hbox {s, 3H}, 3^\prime \hbox {-OCH}_{3}),\, 3.77 \,(\hbox {s, 2H}, 5^\prime \hbox {-CH}_{2}\hbox {N}),\, 3.74 \,(\hbox {s, 6H, 3-OCH}_{3 }\hbox {and 5-OCH}_{3}), 2.66-2.49 \,(\hbox {m, 4H, NCH}_{2}),\, 1.79{-}1.74 \,(\hbox {m, 4H, CH}_{2}); ^{13} \hbox {C NMR }(\hbox {100}\hbox {MHz, CDCl}_{3}): \delta \,159.9,\, 147.1,\, 146.5,\, 138.7,\, 128.2, 127.1, 125.0, 120.4, 118.6, 107.7, 103.3, 98.5, 60.2, 55.0, 54.3, 49.1, 33.0;\hbox { EIMS } m/z{:}\hbox { 369[M]}^{+}$.

#### $(E){\text {-}}3^\prime$*-Methoxyl-5*$^\prime$*-(morpholinomethyl)pterostilbene* (**15**)

**15 ** was obtained as yellow oil (199 mg, yield 74 %). $^{1}\hbox {H NMR }(\hbox {400 MHz, CDCl}_{3}){:}\, \delta \,6.91(\hbox {s, 1H}, 6^\prime \hbox {-H}) 6.90 \,(\hbox {d}, J = \hbox {16.2 Hz, 1H}, \beta \hbox {-CH=}),\, 6.79 \,(\hbox {d}, J = 16.2\hbox { Hz, 1H}, \alpha \hbox {-CH=}),\, 6.69 \,(\hbox {s, 1H}, 2^\prime \hbox {-H}),\, 6.56 (\hbox {d}, J = \hbox {2.1 Hz, 2H, 2-H} \hbox {and 6-H}),\, 6.29 \,(\hbox {t}, J = \hbox {2.1 Hz, 1H, 4-H}),\, 3.85 \,(\hbox {s, 3H}, 3^\prime \hbox {-OCH}_{3}),\, 3.74 \,(\hbox {s, 6H, 3-OCH}_{3}\hbox { and 5-OCH}_{3}),\, 3.68 \,(\hbox {s, 2H}, 5^\prime \hbox {-CH}_{2}\hbox {N}),\, 3.67 \,(\hbox {t}, J = \hbox {4.2 Hz, 4H,} \hbox {NCH}_{2}),\, 2.52 \,(\hbox {s, 4H,} \hbox {OCH}_{2}).^{ 13}\hbox {C NMR }\,(\hbox {100 MHz, CDCl}_{3})\,{:}\, \delta \,160.9, 148.0, 146.9, 139.5, 129.0, 128.4, 126.1, 120.7, 119.7, 108.8, 104.2, 99.5, 66.7, 61.5, 55.8, 55.1, 52.8;\hbox { EIMS }m/z: \hbox { 385 [M]}^{+}$.

#### $(E){\text {-}}3^\prime$*-Methoxyl-5*$^\prime$*-(piperidin-4-ol-1-ylmethyl)pterostilbene* (**16**)

**16 ** was obtained as yellow oil (170 mg, yield 61 %). $^{1}\hbox {H NMR } \,(\hbox {400 MHz, CDCl}_{3}){:} \delta \,6.98 (\hbox {d}, J \!=\! 16.2,\hbox { Hz,} \hbox {1H}, \beta \hbox {-CH=}),\, 6.97(\hbox {s, 1H}, 6^\prime \hbox {-H}),\, 6.86 \,(\hbox {d}, J = \hbox {16.2 Hz, 1H}, \alpha \hbox {-CH=}),\, 6.75 \,(\hbox {d}, J = \hbox {1.2 Hz, 1H}, 2^\prime \hbox {-H}),\, 6.64 \,(\hbox {d}, J = \hbox {2.2 Hz, 2H, 2-H and 6-H}),\, 6.37 \,(\hbox {t}, J = \hbox {2.1 Hz, 1H,4-H}),\, 3.93 \,(\hbox {s, 3H}, 3^\prime \hbox {-OCH}_{3}),\, 3.82 \,(\hbox {s, 6H, 3-OCH}_{3}\hbox { and 5-OCH}_{3}),\, 3.72 \,(\hbox {s, 2H}, 5^\prime \hbox {-CH}_{2}\hbox {N}),\, 3.68{-}3.65(\hbox {m, 1H, CH-O}),\, 2.86{-}2.37 \,(\hbox {m, 4H, NCH}_{2}),\, 1.93{-}1.60 \,(\hbox {m, 4H, 2CH}_{2}); ^{13} \hbox {C NMR }(\hbox {100 MHz, CDCl}_{3}): \delta \,160.8,\, 147.9,\, 147.4,\, 139.6,\, 129.4, 127.9, 125.8, 122.0, 119.2, 108.3,\, 104.1,\, 99.4,\, 58.0,\, 55.8, 55.2, 53.3, 29.8, 23.5;\hbox { EIMS }m/z: 399\hbox {[M]}^{+}$.

### Assay for antiproliferative activity

The CCK-8 reagent solution $(10\,\%, 10\,\upmu \hbox {L})$ was added to each well of a 96-well microtiter plate containing $5\times 10^{3}$ cells (Hela), then different concentrations of compounds **1**–**16**$(100, 25, 6.25, 1.56, 0.39, 0.0976, 0.0244, 0.0061\,\upmu \hbox {M})$ were added and incubated for 48 h [18]. Absorbance was measured at $450\sim 490$ nm using a microplate reader. A dose-response curve was plotted for each compound, and the half maximal inhibitory concentration $(\hbox {IC}_{50})$ for cancer cell lines was recorded.

## Electronic supplementary material

Supplementary material 1 (doc 7094 KB)
