# Supplementary material for: Synthesis and antiproliferative activity of pterostilbene and 3′-methoxy pterostilbene Mannich base derivatives against Hela cells
Source: Mol Divers. 2015 Jul 11;19(4):737–43. doi: 10.1007/s11030-015-9615-1 (PMC4591201; doi:10.1007/s11030-015-9615-1)
Supplement: Supplementary file 1 — Supplementary material 1 (doc 7094 KB) [file 11030_2015_9615_MOESM1_ESM.doc]

**Synthesis and Antiproliferative Activity of Pterostilbene and** **3′-Methoxy Pterostilbene Mannich Base Derivatives Against Hela Cells**

Liu Chongyang, Dong Linpei, Wang Shengchun, Wang Qiuan*

College of Chemistry and Chemical Engineering,Hunan University, Changsha 410082, P.R.China

**^1^HNMR ^13^CNMR and MS spectral data for Compound 3~16 and the dose-response curve for compound 1-16**

Explanation：

^1^HNMR spectra phenolic signals of Compound **6, 8, 11, 12, 13, 14, 15, 16** were missing because the peak of phenolic appears short and wide，or be covered by the line of the solvent，so we cann′t find the signals.

**Compound 3 ^1^HNMR**

**
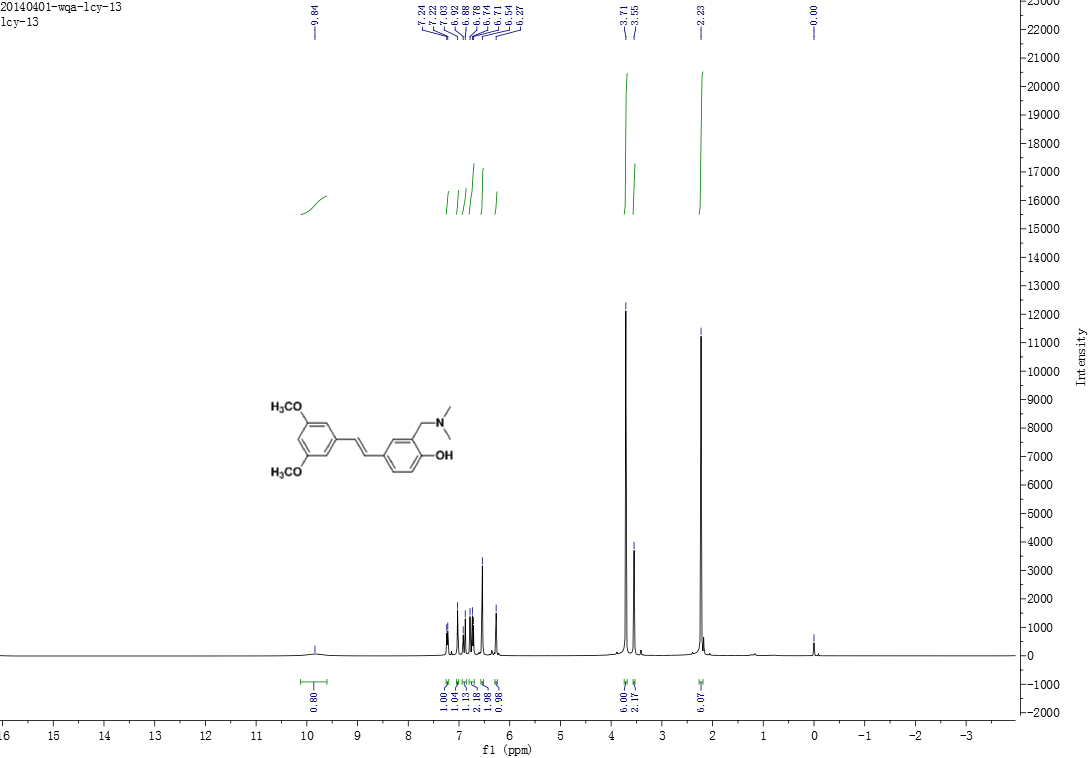
**

**Compound 3 ^13^CNMR**

**
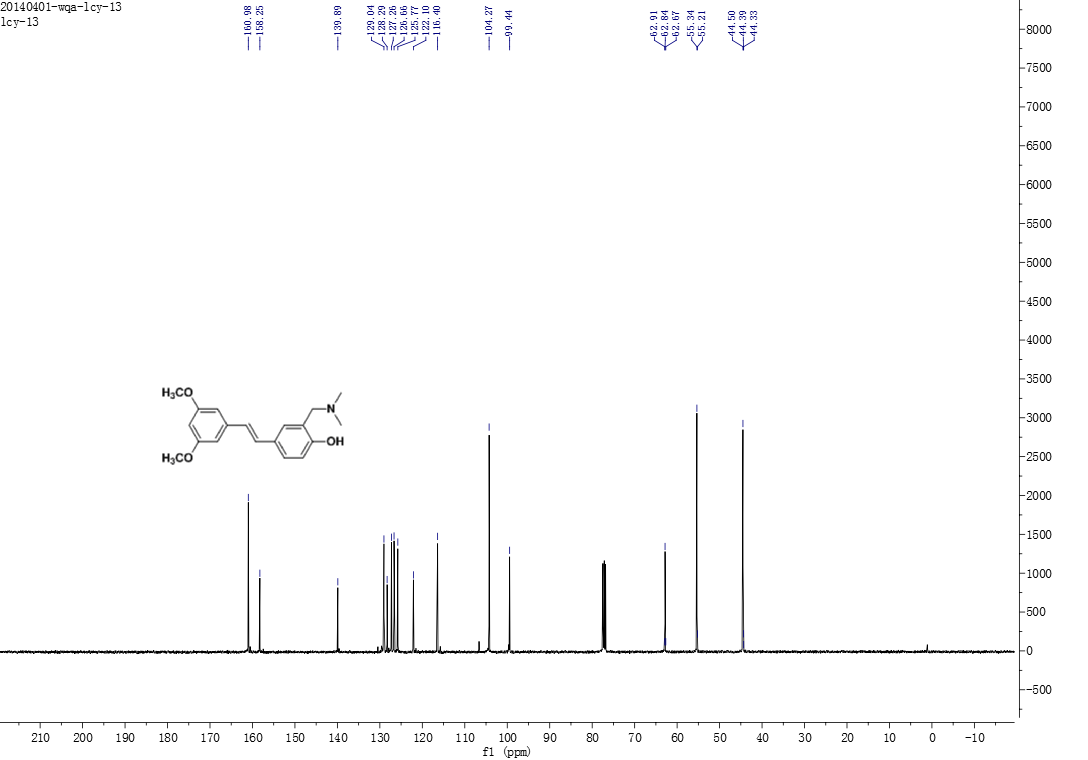
**

**Compound 3 ESIMS**

**Compound 4 ^1^HNMR**

**
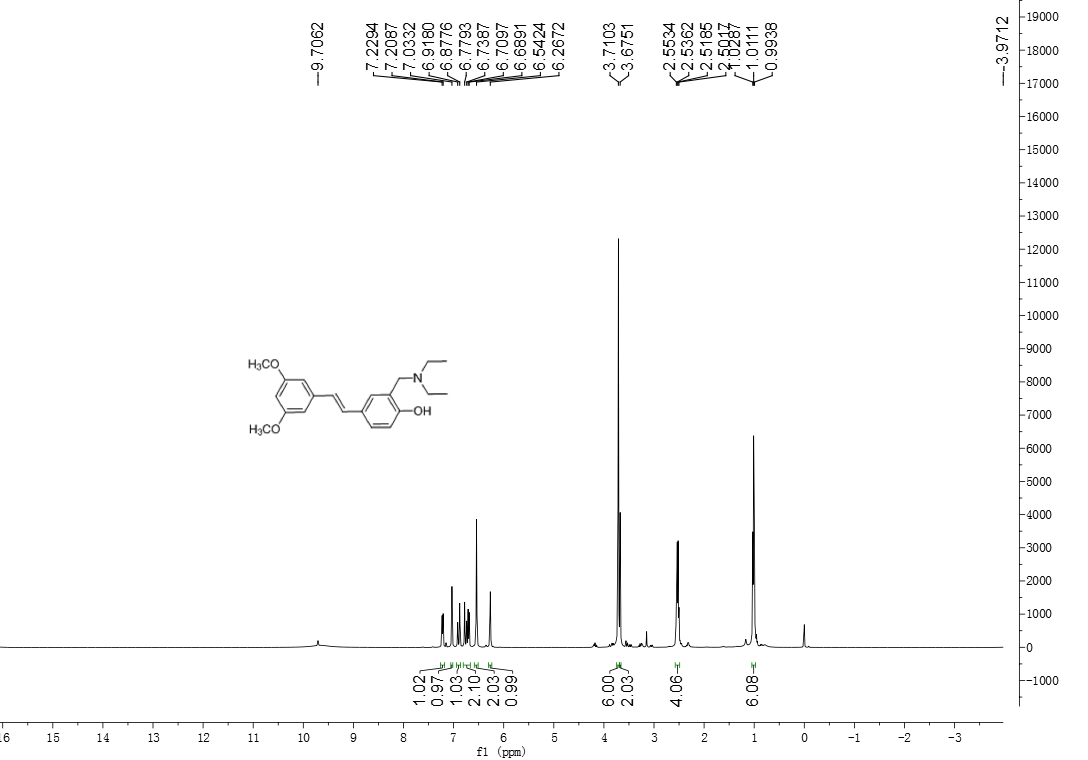
**

**Compound 4 13CNMR**


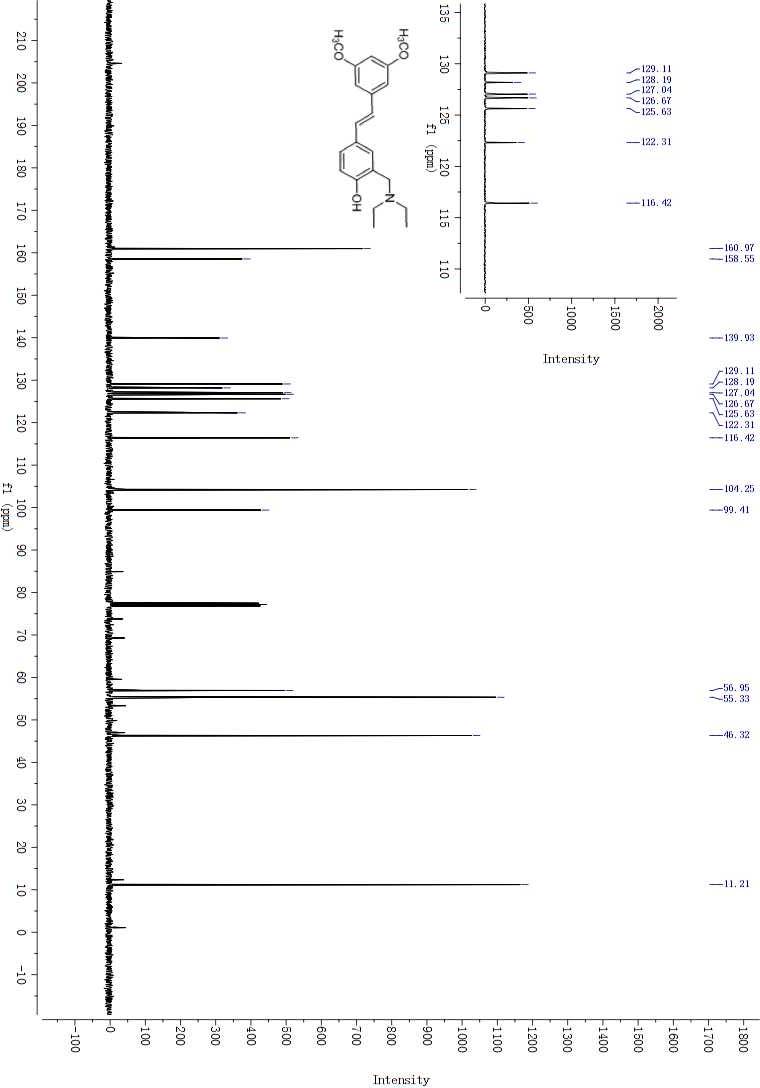


**Compound 4 ESIMS**

**Compound 5 ^1^HNMR**

**
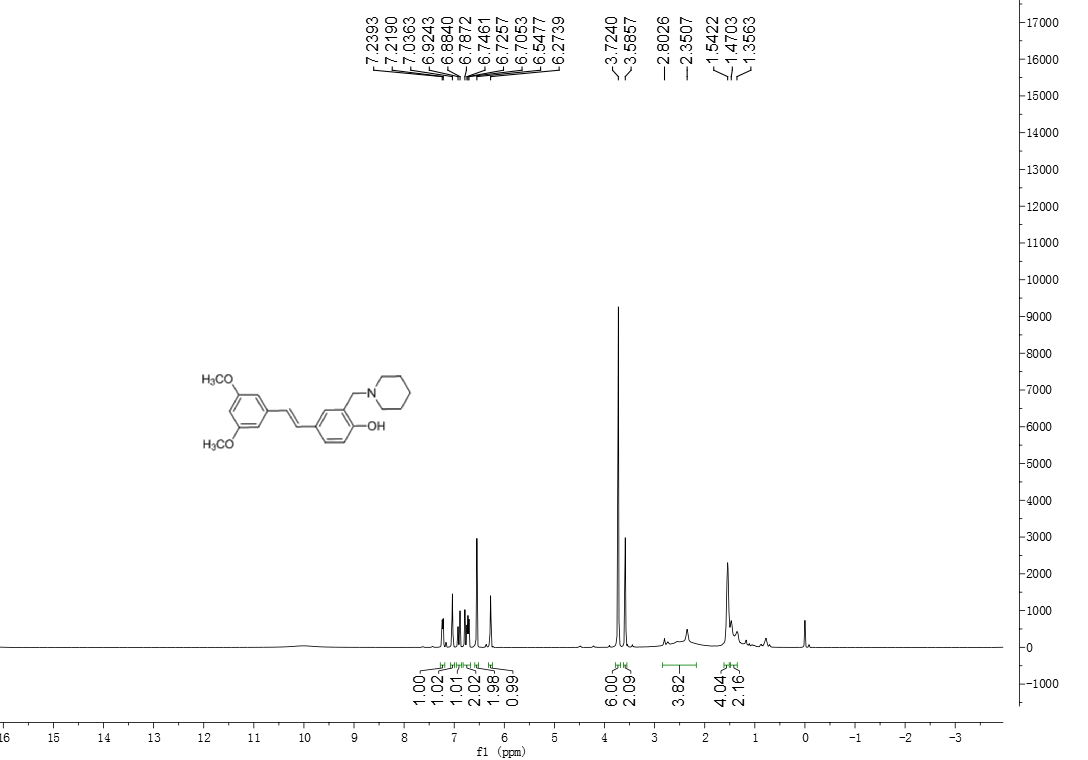
**

**Compound 5 13CNMR**

**
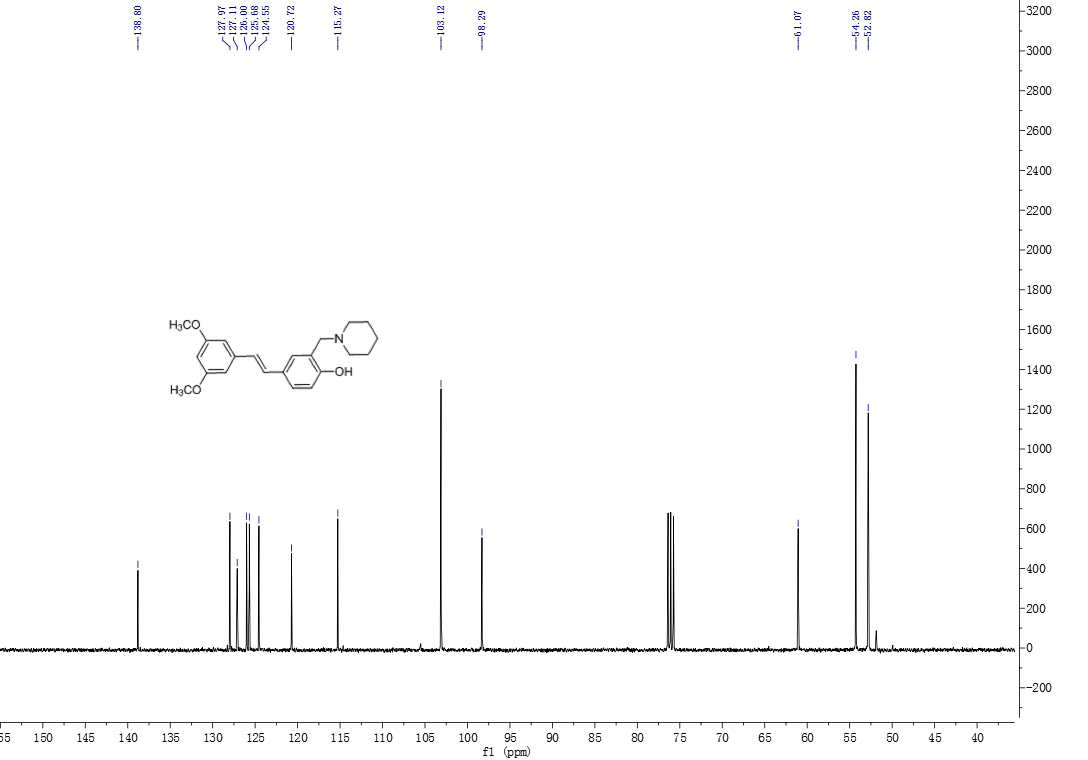
**

**Compound 5 EIMS**

**Compound 6 ^1^HNMR**

**
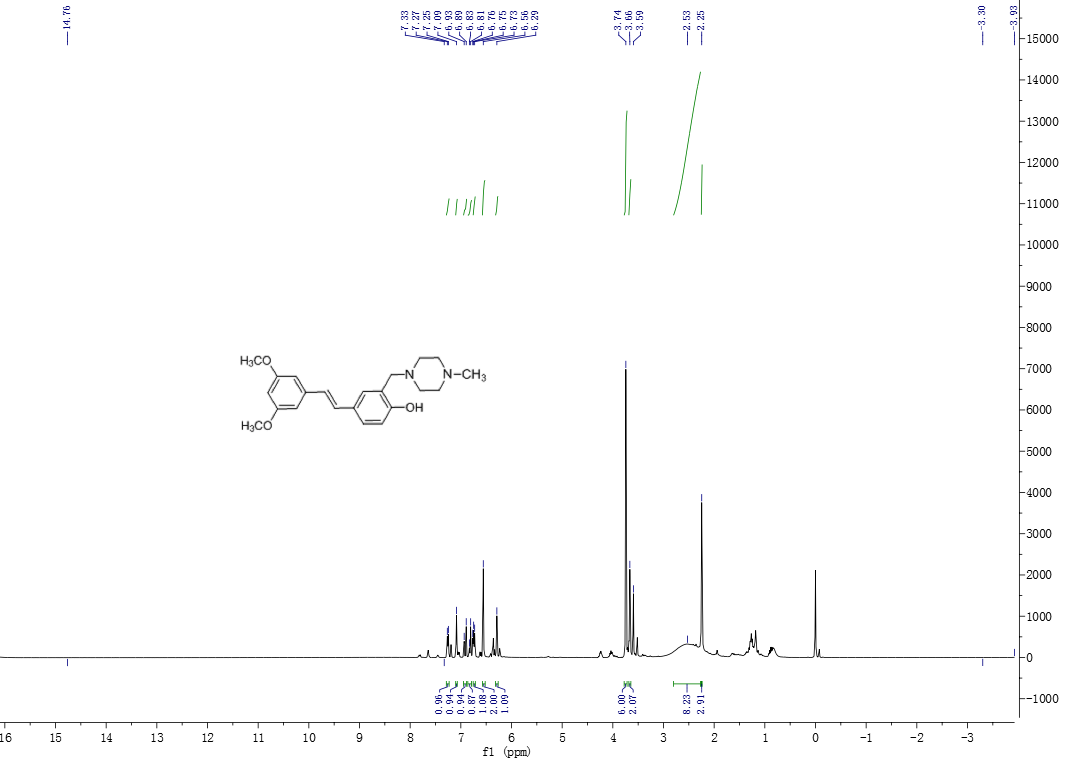
**

**Compound 6 ^13^CNMR**

**
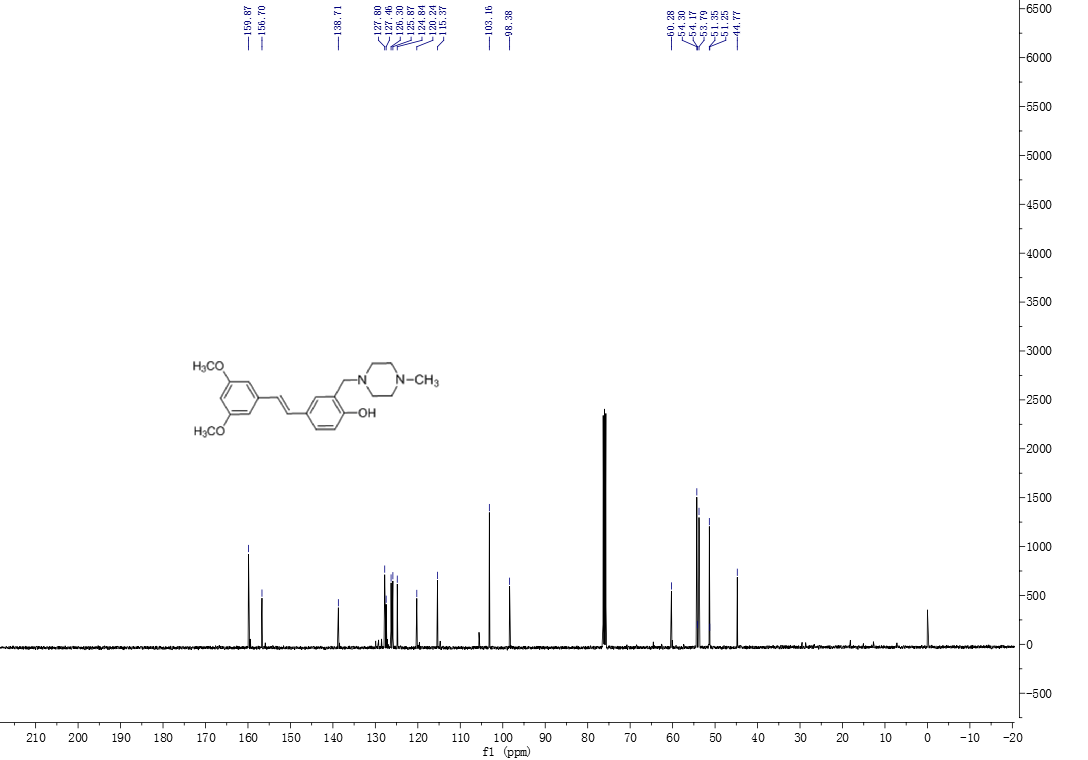
**

**Compound 6 ESIMS**

**Compound 7 ^1^HNMR**

**
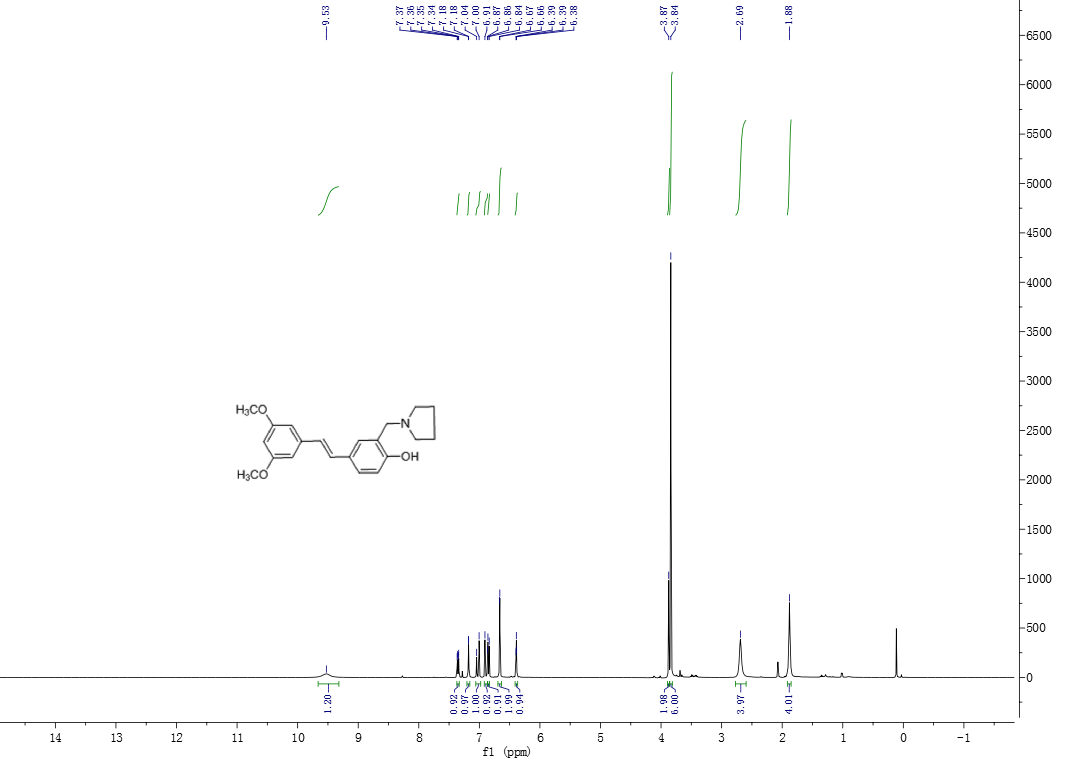
**

**Compound 7 ^13^CNMR**

**
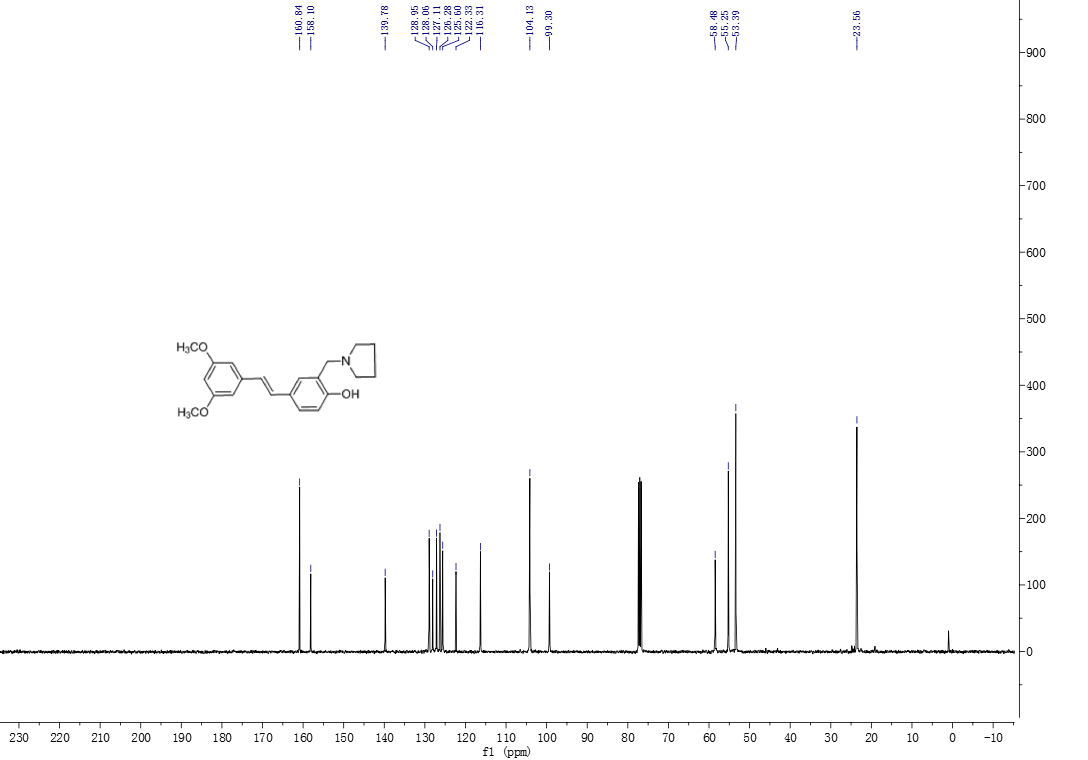
**

**Compound 7 ESIMS**

**Compound 8 ^1^HNMR**

**
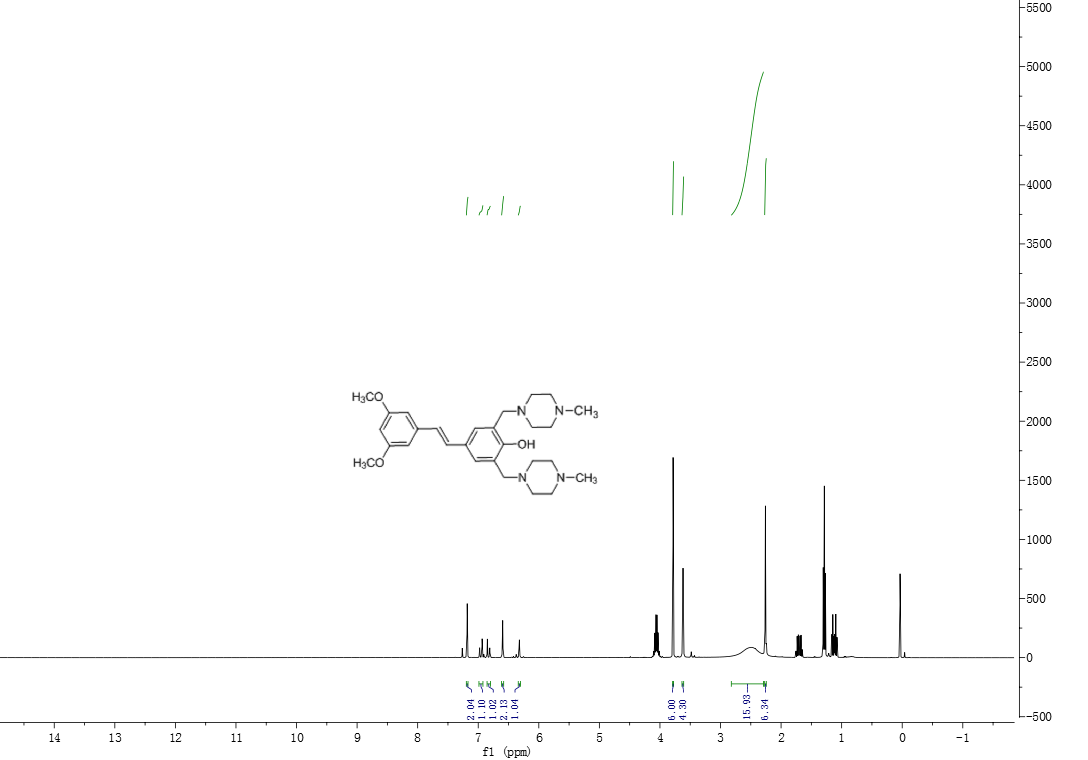
**

**Compound 8 ^13^CNMR**

**
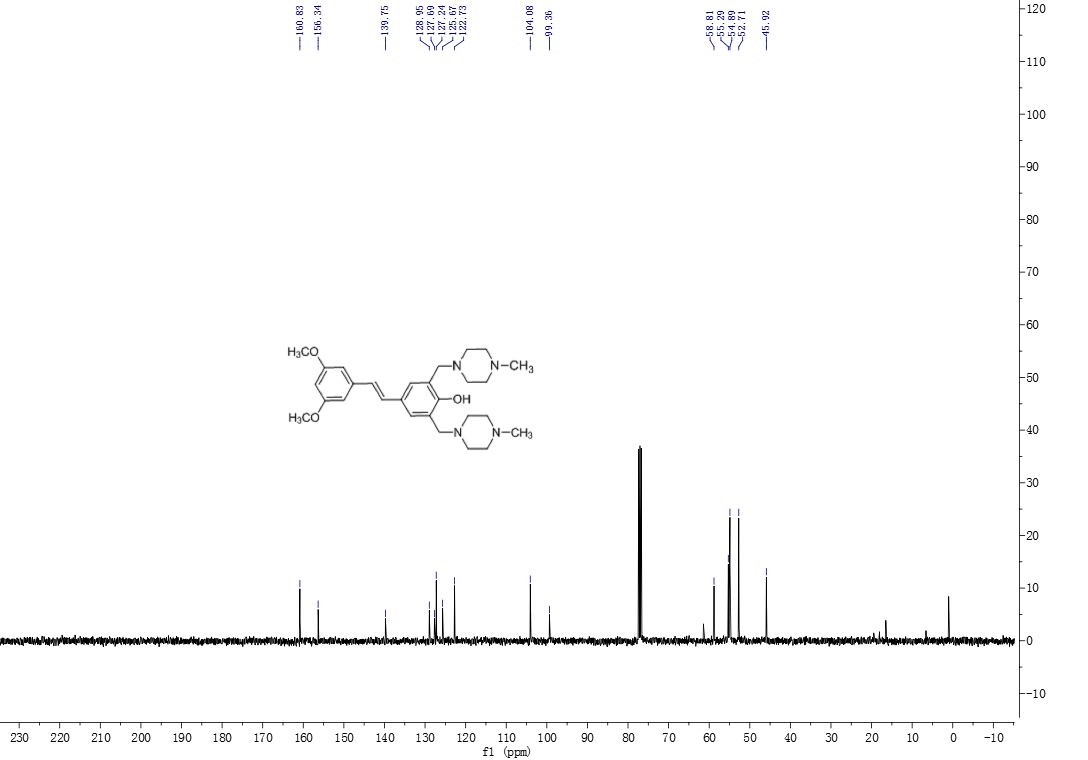
**

**Compound 8 ESIMS**

**Compound 9 ^1^HNMR**

**
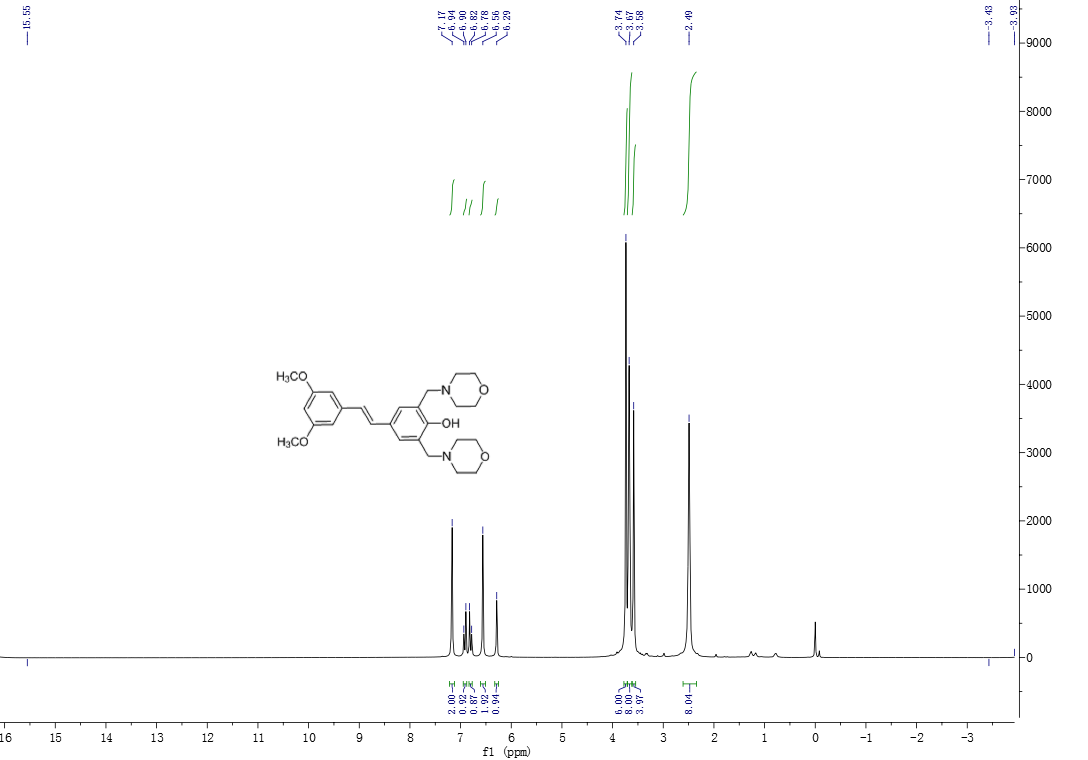
**

**Compound 9 ^13^CNMR**

**
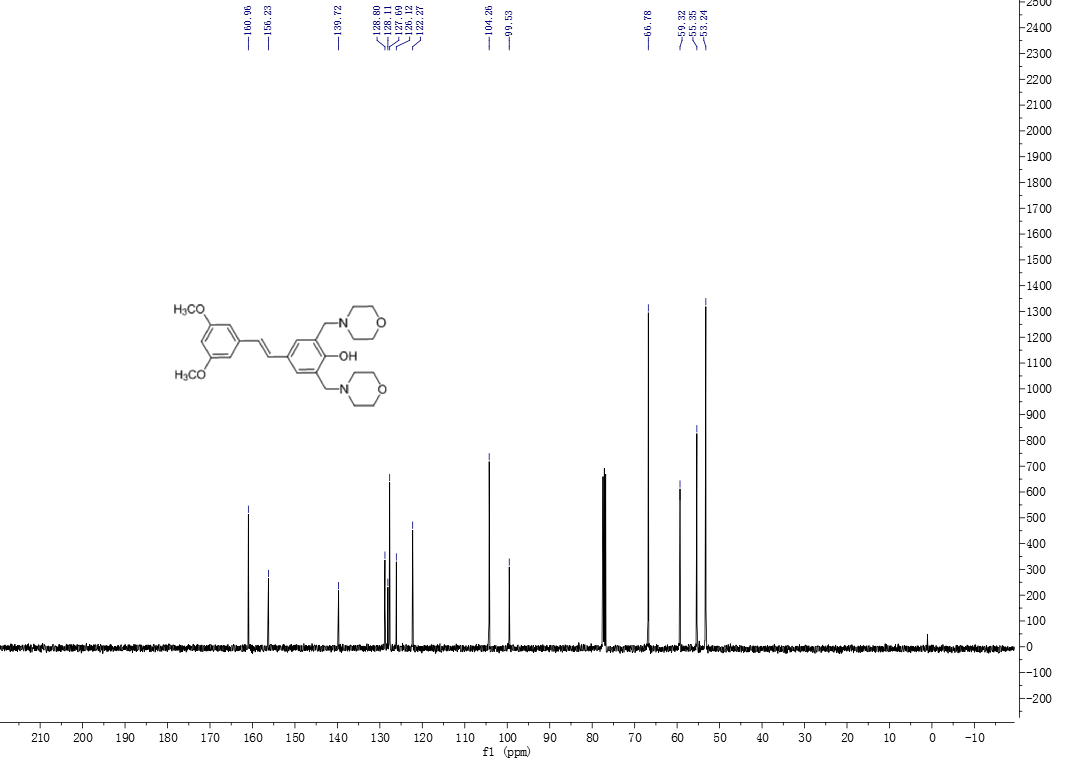
**

**Compound 9 ESIMS**

**Compound 10 ^1^HNMR**

**
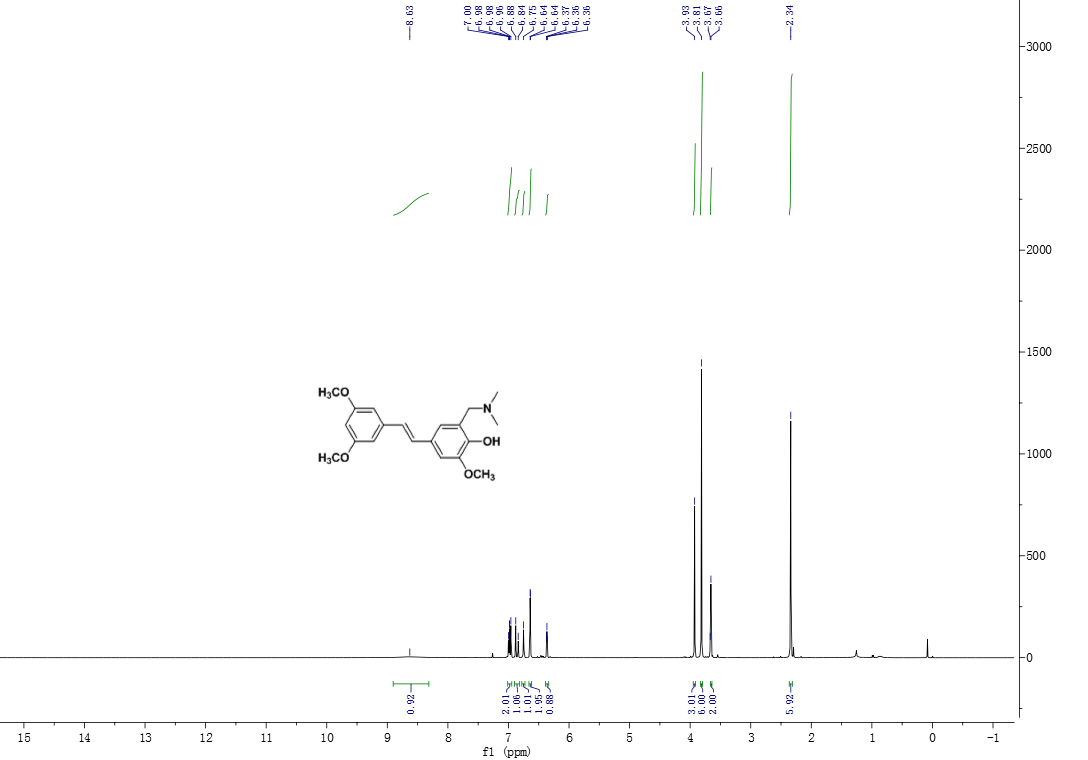
**

**Compound 10 ^13^CNMR**

**
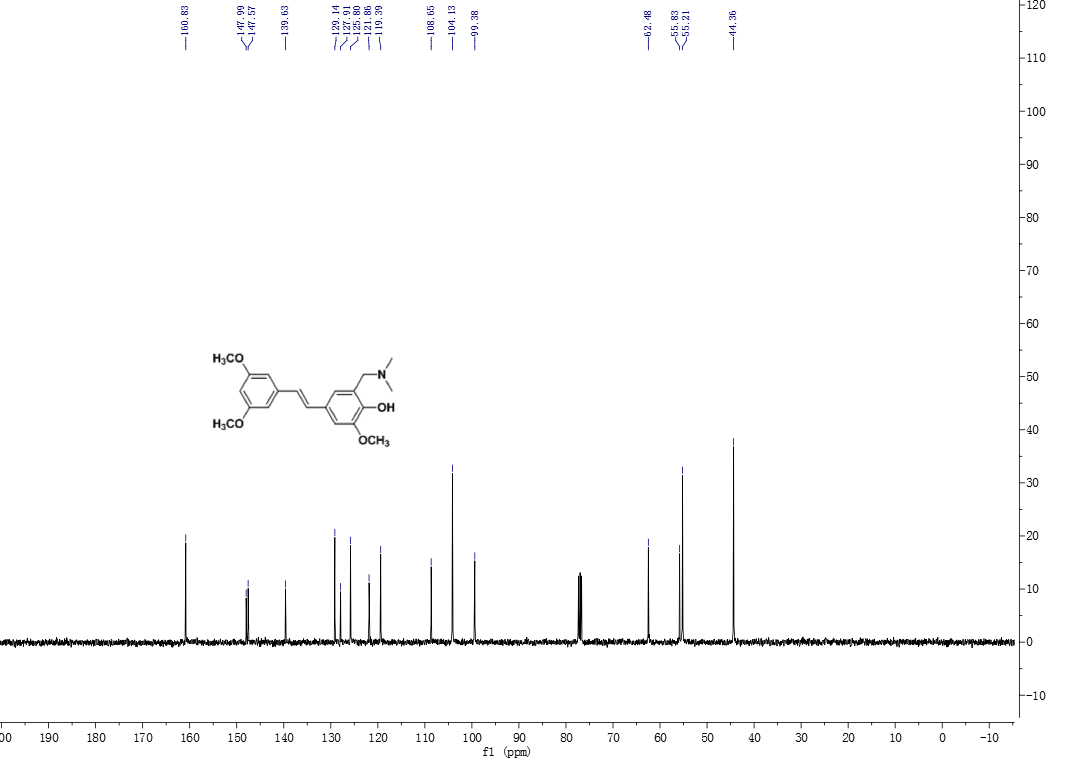
**

**Compound 10 EIMS**

**Compound 11 ^1^HNMR**

**
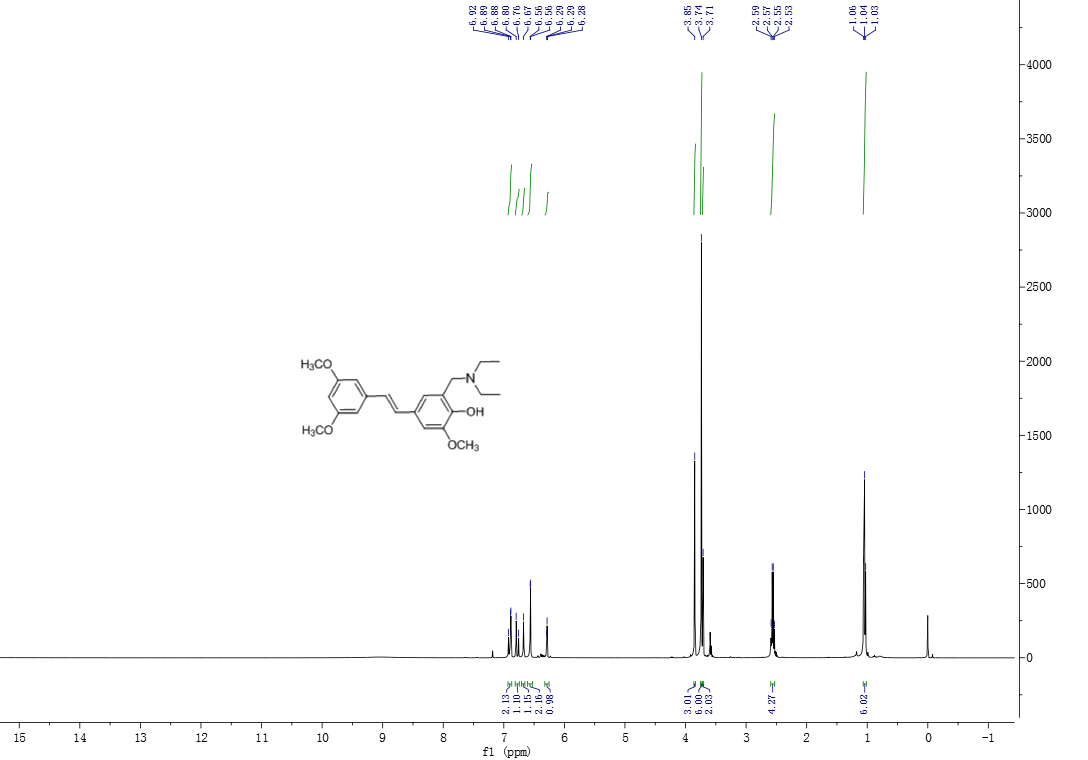
**

**Compound 11 ^13^CNMR**

**
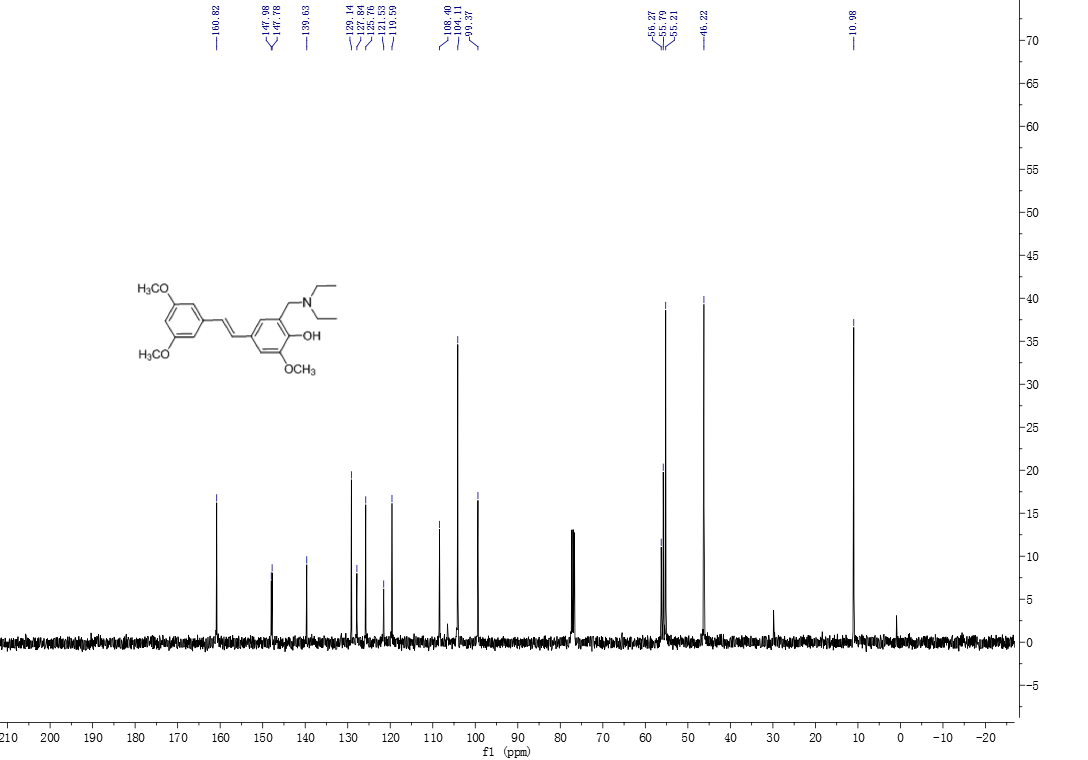
**

**Compound 11 EIMS**

**Compound 12 ^1^HNMR**

**
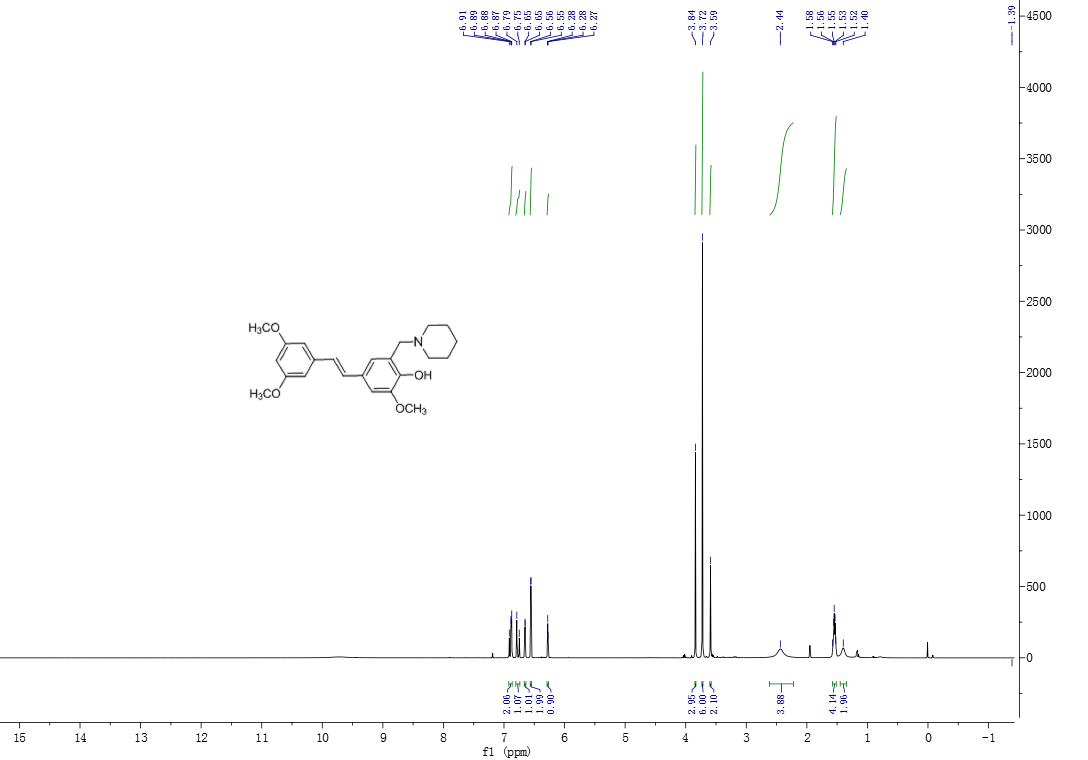
**

**Compound 12 ^13^CNMR**

**
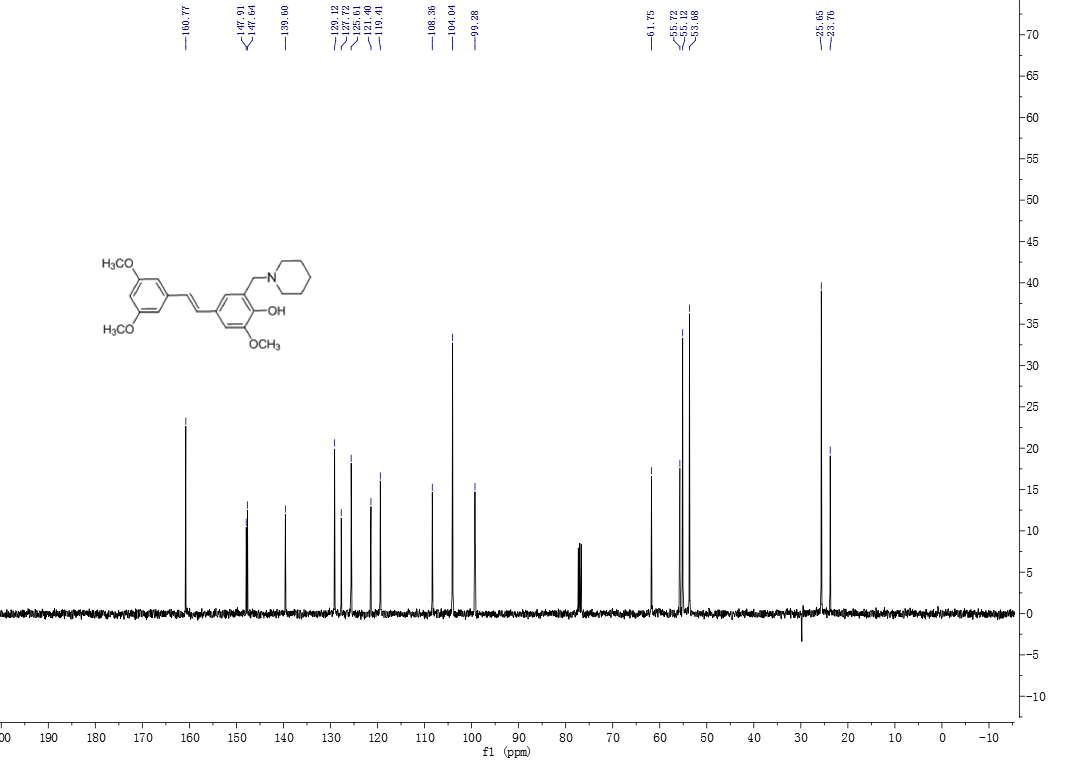
**

**Compound 12 EIMS**

**Compound 13 ^1^HNMR**

**
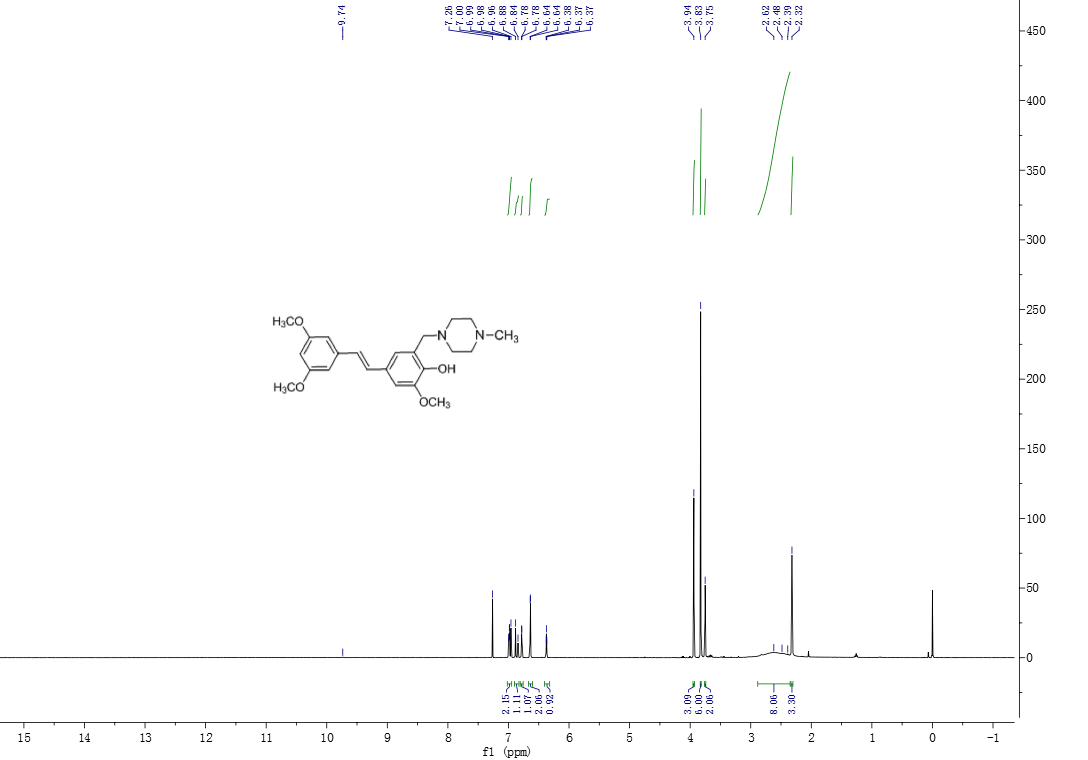
**

**Compound 13 ^13^CNMR**

**
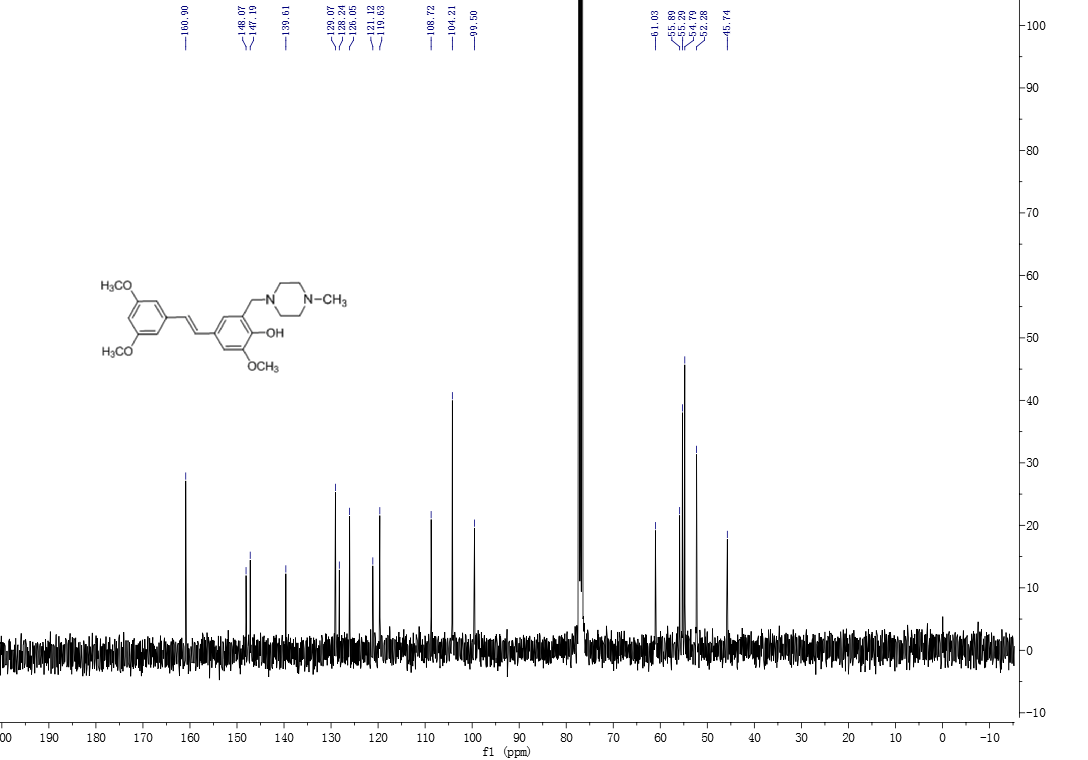
**

**Compound 13 EIMS**

**Compound 14  ^1^HNMR**

**
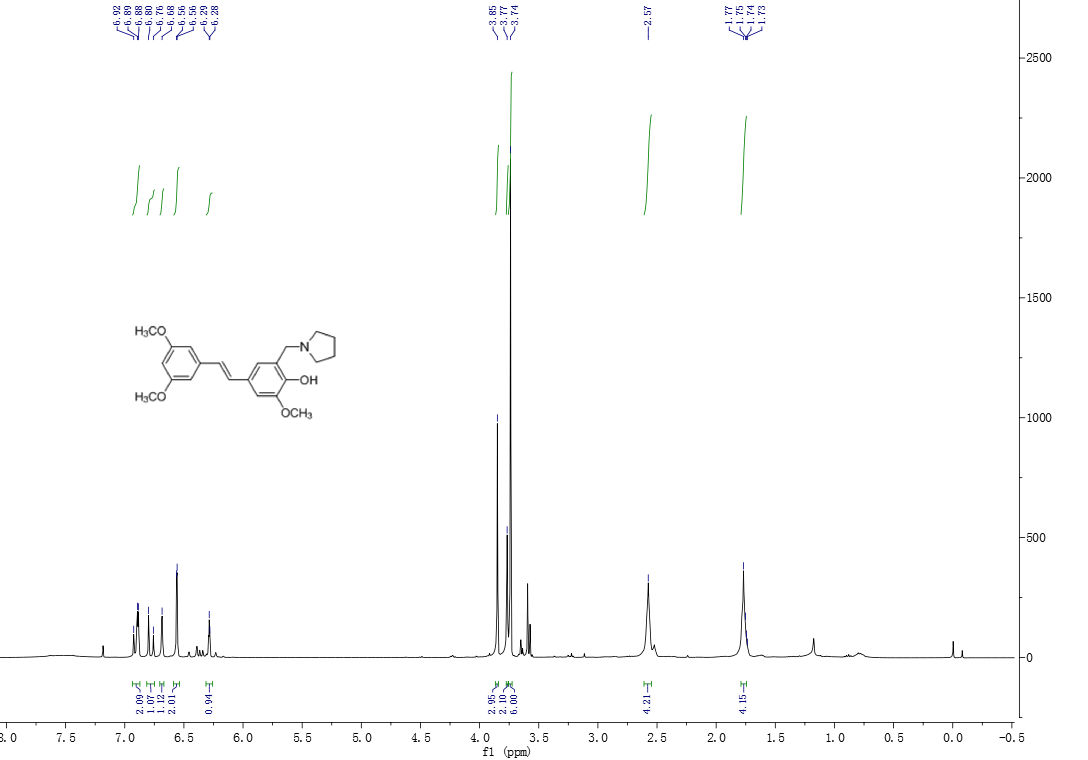
**

**Compound 14 ^13^CNMR**

**
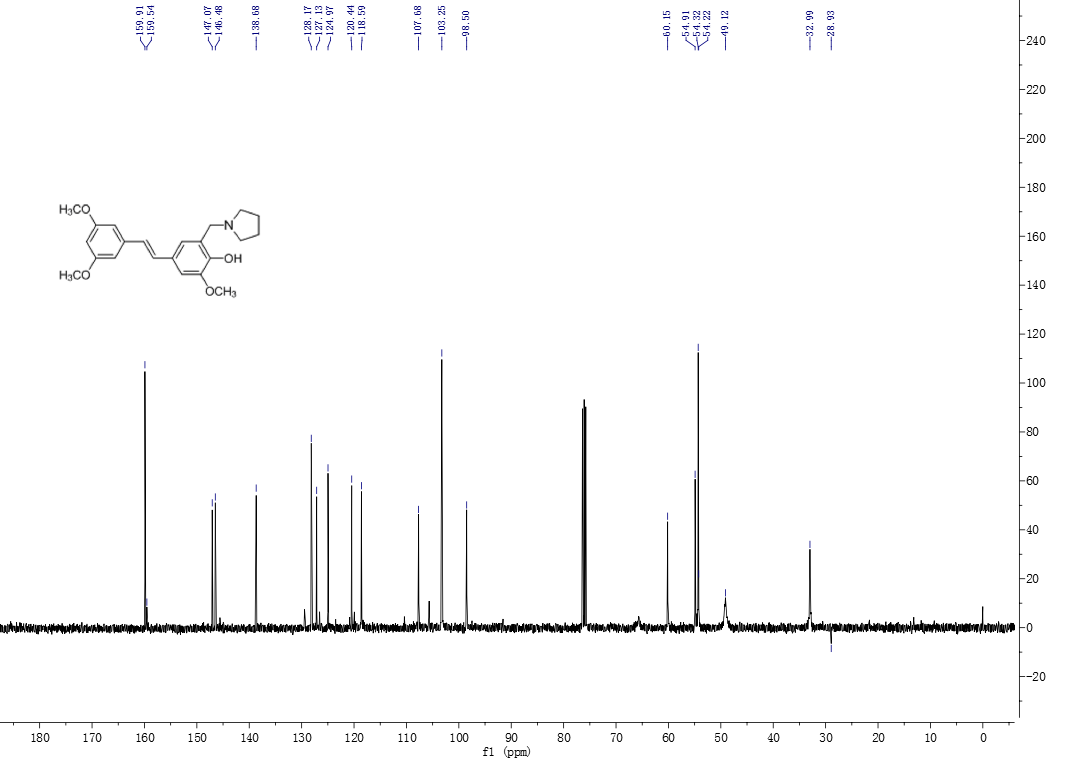
**

**Compound 14 EIMS**

**Compound 15 ^1^HNMR**

**
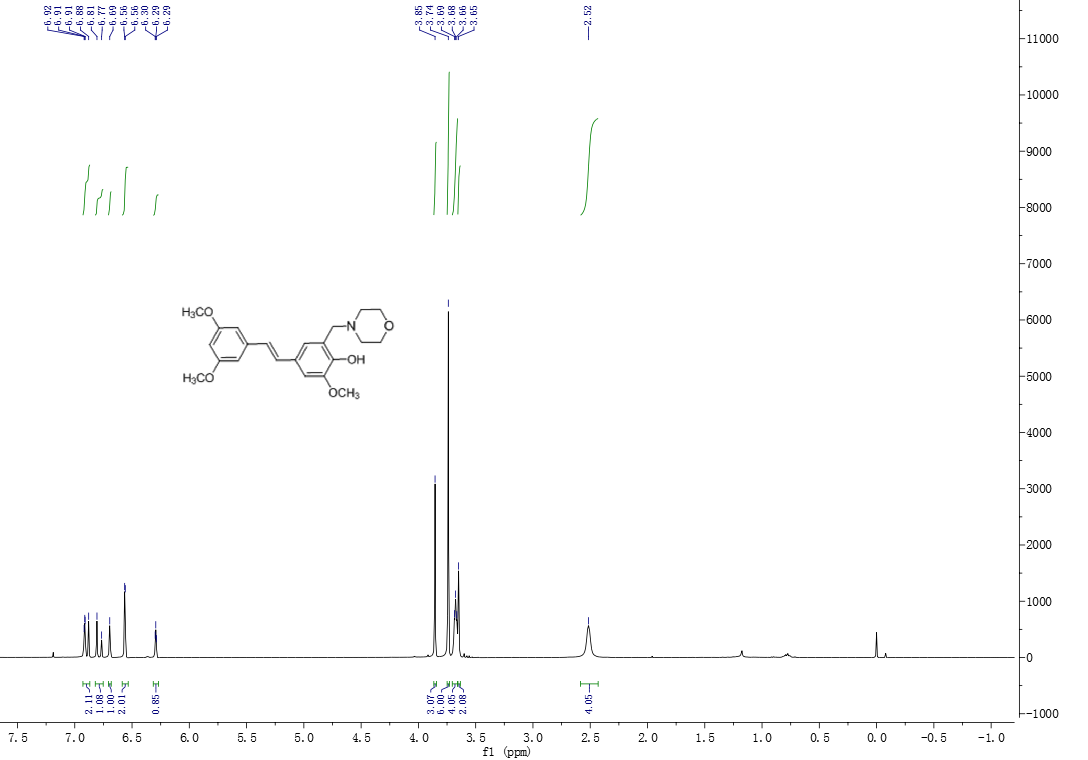
**

**Compound 15 ^13^CNMR**

**
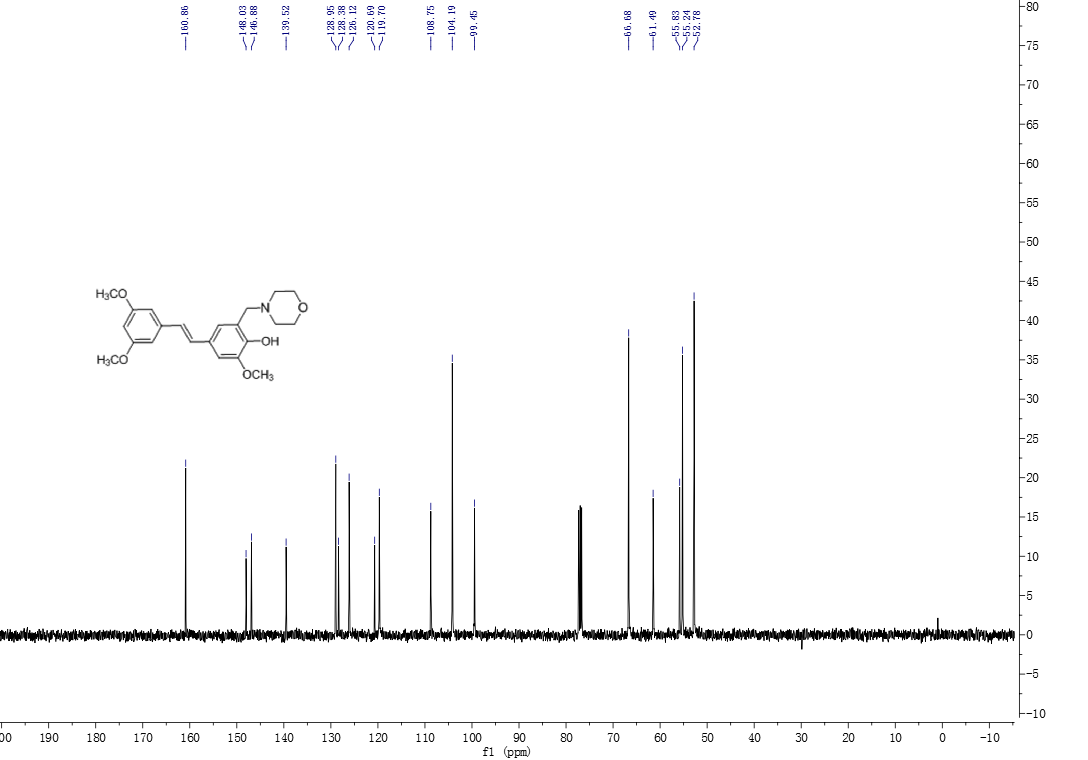
**

**Compound 15 EIMS**

**Compound 16 ^1^HNMR**

**
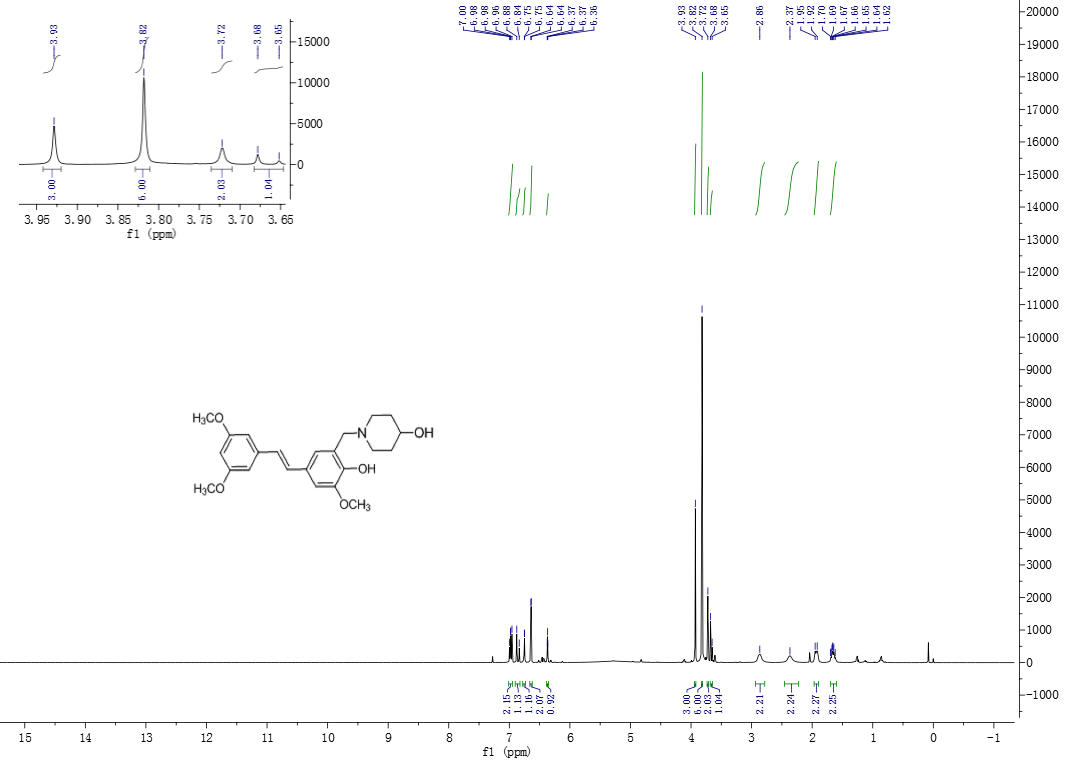
**

**Compound 16 ^13^CNMR**

**
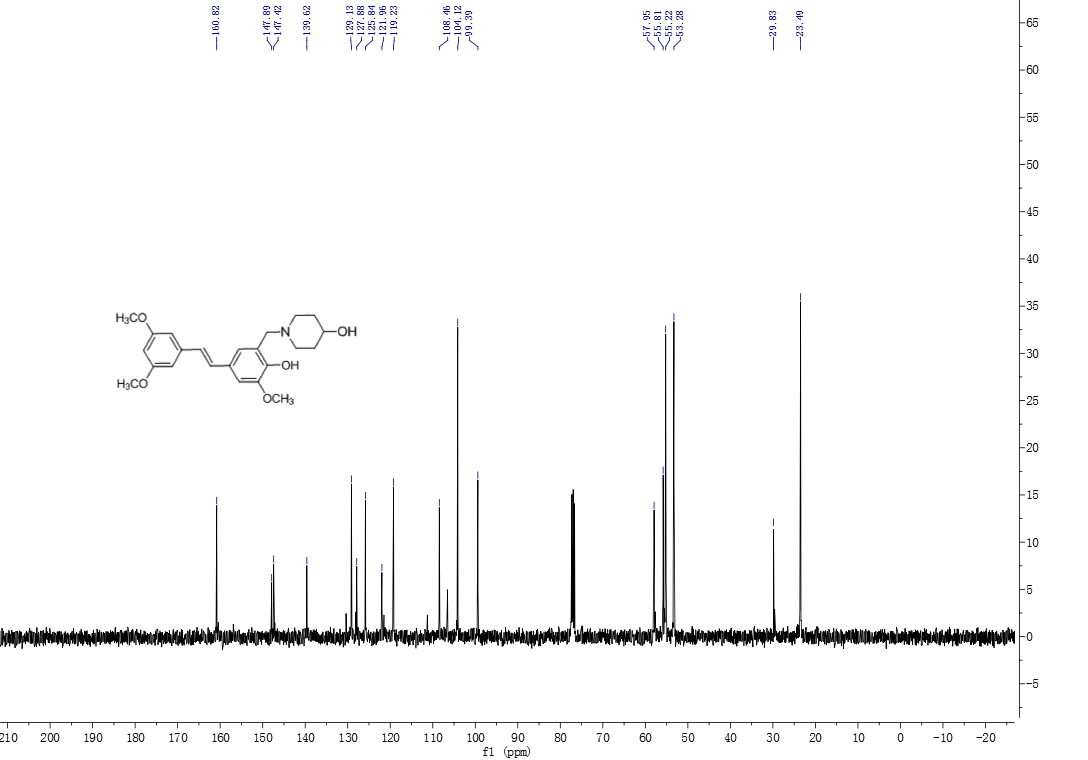
**

**Compound 16 EIMS**

**The dose-response curve for Compound 1-16**
